# Synthesis, characterization and in silico studies of novel multifunctional imidazole-thiazole hybrids with potent antimicrobial and anticancer properties

**DOI:** 10.1038/s41598-025-93249-1

**Published:** 2025-03-21

**Authors:** Deepika Dwarakanath, Yogeesha N. Nayak, Ananda Kulal, Samyak Pandey, K. Sreedhara Ranganath Pai, Santosh L. Gaonkar

**Affiliations:** 1 Department of Chemistry, Manipal Institute of Technology, Manipal Academy of Higher Education, Manipal, 576104 Karnataka India; 2 Biological Sciences Division, Poornaprajna Institute of Scientific Research, Bangalore, 562110 Karnataka India; 3 Department of Pharmacology, Manipal College of Pharmaceutical Sciences, Manipal Academy of Higher Education, Manipal, 576104 Karnataka India

**Keywords:** Imidazole-thiazole derivatives, Synthesis, Antimicrobial activity, Cytotoxicity studies, Molecular docking, Molecular dynamics (MD) simulation, Biochemistry, Cancer, Computational biology and bioinformatics, Chemistry

## Abstract

Treating infections remains a significant challenge, driving the ongoing pursuit of novel drug candidates. Heterocyclic compounds, such as those containing imidazole and thiazole rings, are well-known for their diverse therapeutic and pharmaceutical applications. In this study, we designed, synthesized, and characterized a series of six novel compounds incorporating these two five-membered rings. The synthesis involved the reaction of different phenacyl bromides with imidazole-hydrazinecarbothioamide to produce imidazole-thiazole hybrid derivatives, which were confirmed through IR, ^1^H NMR, ^13^C NMR, and mass spectrometry analyses. The antimicrobial activities of the derivatives were evaluated against three bacterial strains and one fungal strain using the serial dilution method, with their minimum inhibitory concentrations (MICs) determined. Notably, all the derivatives exhibited moderate antimicrobial activity. Cytotoxicity assessment revealed that derivative **5a** was particularly excellent, displaying significant inhibition with an IC_50_ value of 33.52 μM. Furthermore, molecular docking, ADME, and molecular dynamics simulations were conducted, focusing on the interaction between derivative **5a** and the protein (PDB ID: 6LUD) to elucidate the stability of the interaction.

## Introduction

**Fig. 1:**
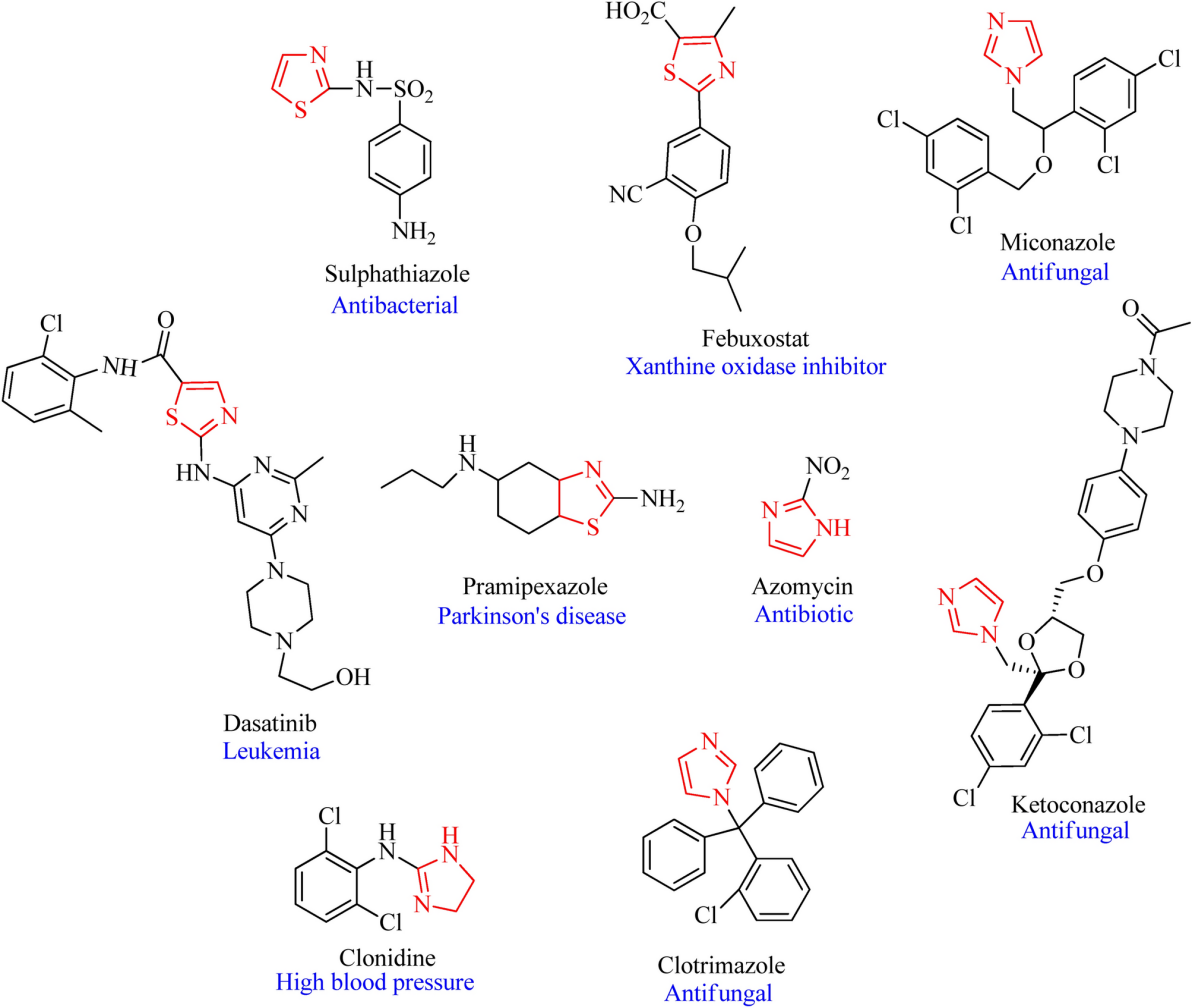
Drugs with thiazole or imidazole scaffolds used as therapeutics.

Most of the active pharmaceutical intermediates or drugs constitute heterocycles, more specifically, nitrogen-containing heterocycles, at times in combination with oxygen or sulfur. When present in five- or six-membered rings, these heteroatoms are superior, contributing to 85% of the biologically active scaffolds^1^. For instance, imidazoles are five-membered, planar, and aromatic, with two nitrogen atoms in their ring system. Structures with this nucleus are well known for various biological activities, such as antimicrobial^2^, anticancer^3,4^, antitubercular^5,6^, and anti-inflammatory^7^; some of these structures are marketed as drugs and used to treat infections^8,9^. Another name of imidazole is 1,3-diazole, which is an important core of natural products such as purines, histamines and histidines. The imidazole ring is soluble in polar solvents and water. Due to its polarity, this structure can help increase the solubility of drugs, making it useful for therapeutic applications^10^. Similarly, thiazoles, which are composed of five-membered rings with nitrogen and sulfur in their ring system, have been extensively explored in medicinal and organic chemistry. Thiazole rings are present in many natural products and in marketed drugs, such as penicillin-G, bleomycin, febuxostat, sulfathiazole, and vitamin B; as a result, they play a key role in the discovery of drugs^11^. Prominent pharmaceutical activities of the scaffold include anticancer^12,13^, anticonvulsant^14^, antimicrobial^15,16^, antidiabetic^17,18^, and anti-inflammatory^19^ activities. In addition to their biological applications, thiazoles also have applications as dyes, photosensitizers and pigments due to their strong S–C–N fragment. They also possess an odor that is nut-like from cocoa extract and is one of the constituents of the current food industry^20^. Marketed drugs with thiazole and imidazole scaffolds are represented in Fig. 1^2,21^.

**Fig. 2:**
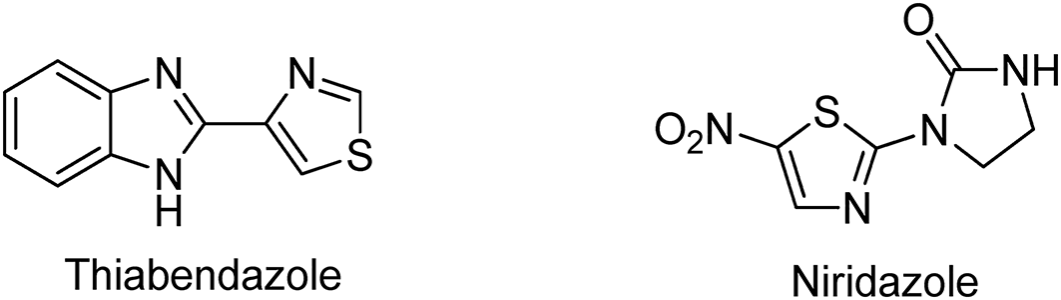
Drugs containing imidazole and thiazole scaffolds.

Thus, the combination of the two heterocycles, imidazole and thiazole, in one structure may result in increased therapeutic action. Drugs like thiabendazole, an antihelminthic drug, and niridazole, used in the treatment of schistosomiasis, which are available on the market, contain imidazole and thiazole rings in their skeletons, as shown in Fig. 2. In this study, we synthesized and characterized a series of six novel imidazole-thiazole hybrids and evaluated their antimicrobial and anticancer activities. Additionally, in silico studies were conducted to further investigate the ligand–protein interactions.

## Results and discussion

### Chemistry

**Scheme 1:**
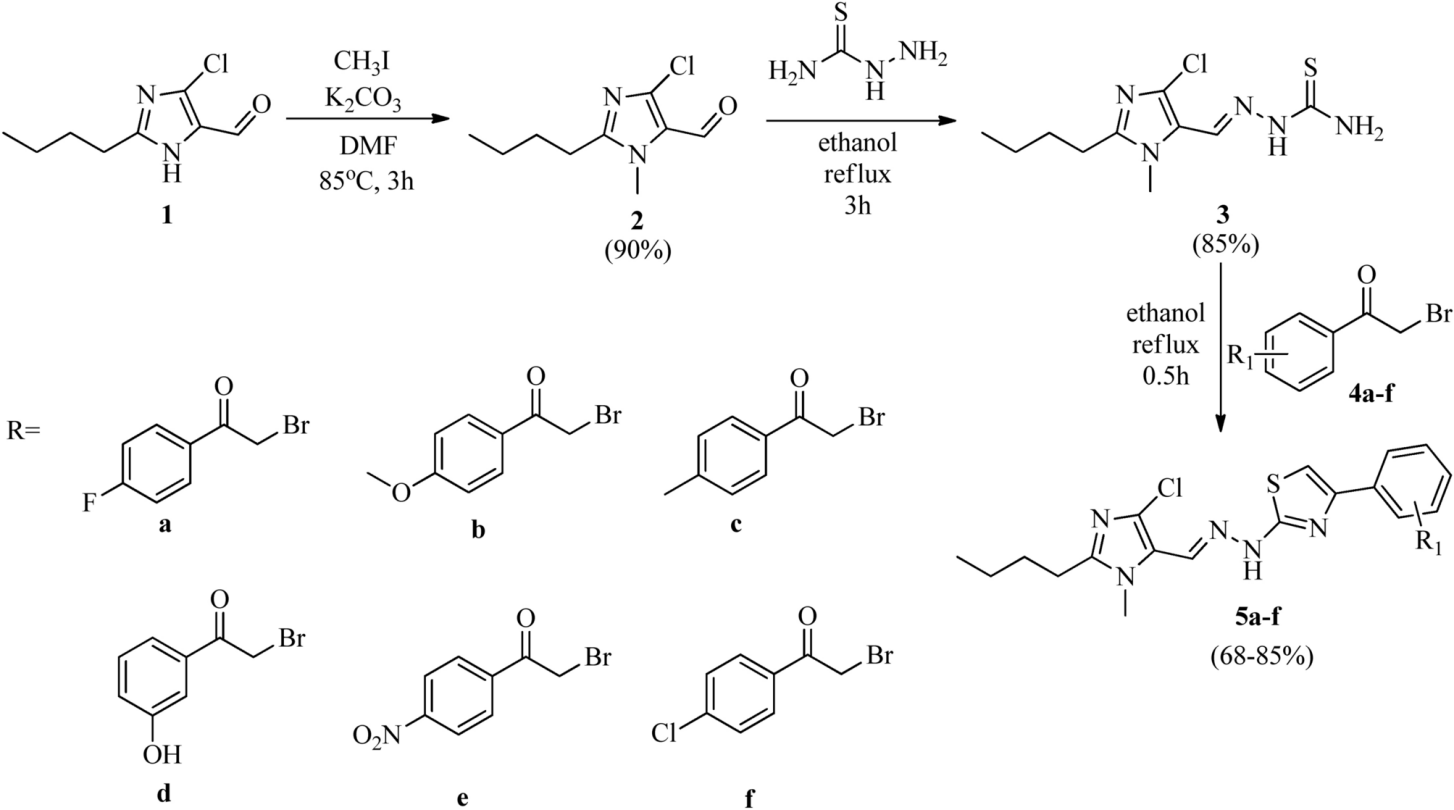
Synthetic route for imidazole-thiazole hybrids.

The schematic route for the synthesis of the target compounds is represented in Scheme 1. Initially, the imidazole-aldehyde was methylated to protect the nitrogen from any further nucleophilic attack in the last step. The alkylated aldehyde was then reacted with thiosemicarbazide, followed by cyclization to obtain the thiazole ring.

**Scheme 2:**
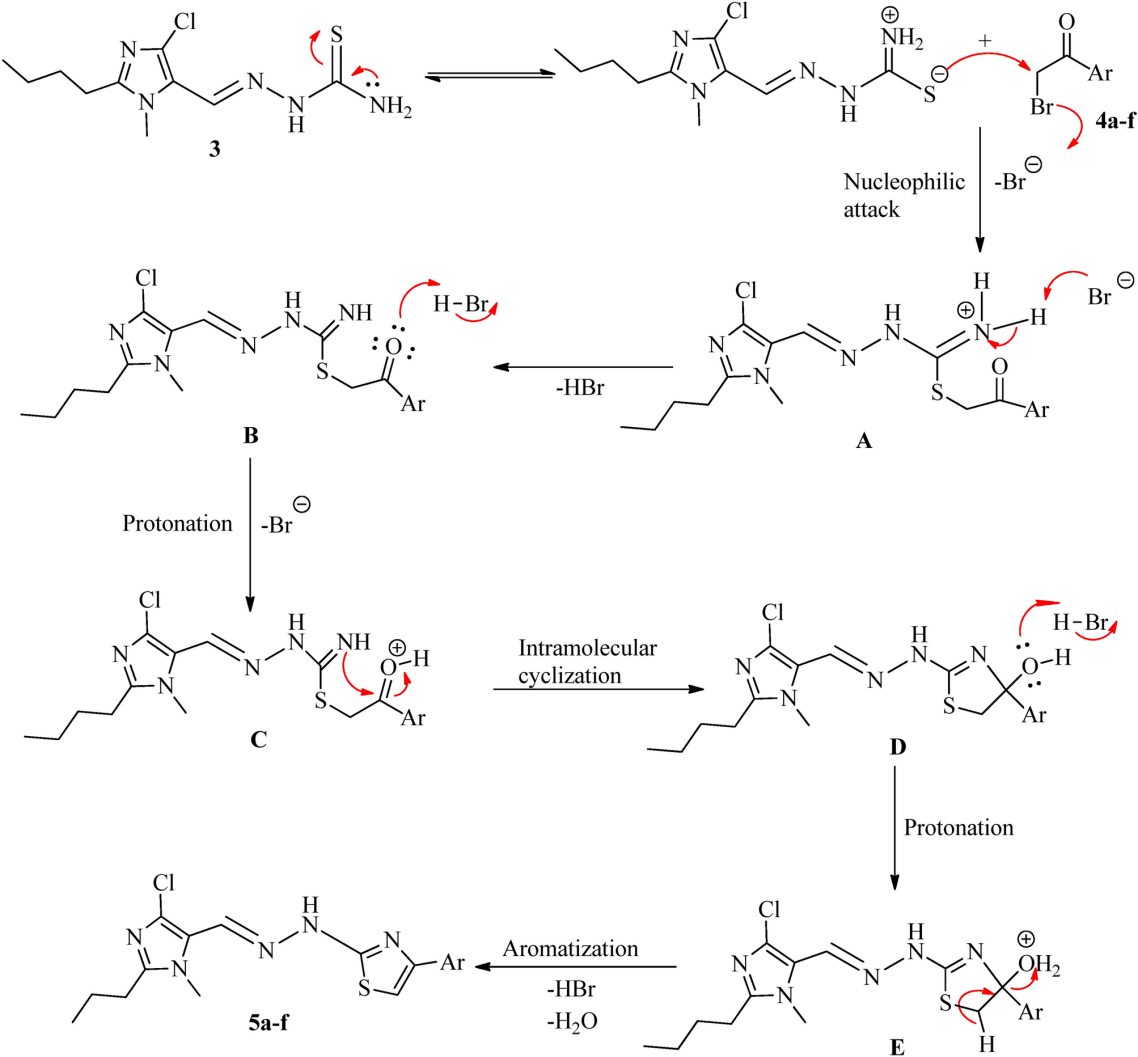
Probable mechanism for the formation of the thiazole ring.

Scheme 2 illustrates the probable mechanism for the formation of the thiazole ring. Phenacyl bromides and thiosemicarbazone easily interact through nucleophilic attack, resulting in **A**. Intermediate **B** is formed upon proton abstraction; followed by activation of the carbonyl group through protonation, which results in **C**. This undergoes intramolecular cyclization followed by aromatization through removal of water and HBr, resulting in thiazoles^22^. The synthesized imidazole and thiazole hybrids were confirmed using IR, proton, ^13^C NMR and mass spectrometry.

The IR data revealed the C=N absorption bands at approximately 1580–1628 cm^−1^, and the C=C aromatic stretching band was in the range of 1444–1490 cm^−1^. The NH peak is present in almost all compounds around the region 3100–3500 cm^−1^. C–N bands are observed in the range of 1269–1279 cm^−1^. The absorption bands around 2900–3000 cm^−1^ represent the aliphatic and aromatic –CH stretching frequencies. Derivative **5b**, bearing a methoxy substituent, shows C-O absorption peak at 1249 cm^−1^, **5d** shows an –OH absorption peak at 3389 cm^−1^, and an absorption peak at approximately 1515 cm^−1^ is observed for the NO_2_ group in derivative **5e**. The ^1^H-NMR spectra confirmed the formation of the derivatives by showing peaks in the aromatic region at 7.09–7.99 ppm. The singlet proton peak at 3.83 ppm corresponds to the methyl protons that were methylated in the first step. The peaks at 0.92–2.69 ppm represent the aliphatic region present in the imidazole ring. The –NH proton peak in some derivatives is not observed, possibly owing to rapid exchange of protons due to the presence of water in the solvent. The ^13^C NMR also confirmed the formation of target products, as the number of carbon peaks corresponded to the number of carbon atoms present in the structure. The mass spectra of the derivatives were analyzed, and the results were consistent with the theoretical calculations.

### Molecular docking

The protein chosen for antibacterial activity is associated with the initiation of fatty acid biosynthesis. Inhibition of this enzyme leads to reduced proliferation, increased cell death and the induction of neural differentiation. For antifungal activity, the ergosterol biosynthesis route was targeted; this sterol is the primary sterol in fungal cell membranes, and because it is essential for protein function, fluidity, and permeability, its suppression results in the termination of life processes. The epidermal growth factor receptor (EGFR) synchronizes the development of epithelial tissue and homeostasis. When this receptor is triggered due to point mutations or upregulation, it causes cancer, mainly related to the lung and breast, and hence is a common target in medicinal chemistry for anticancer activity. Therefore, these proteins were chosen for the current study.

#### Molecular docking with 5BNS

**Fig. 3:**
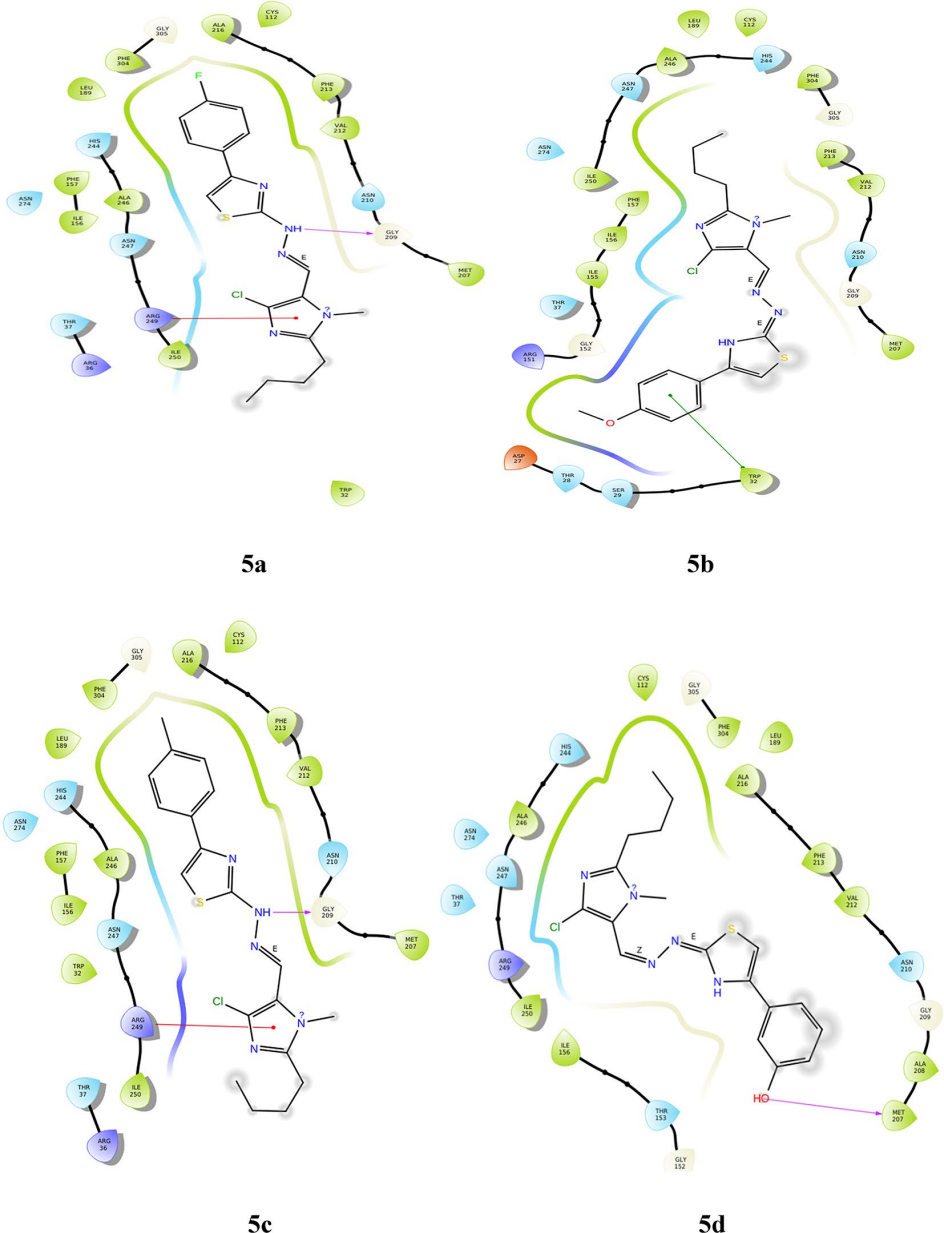

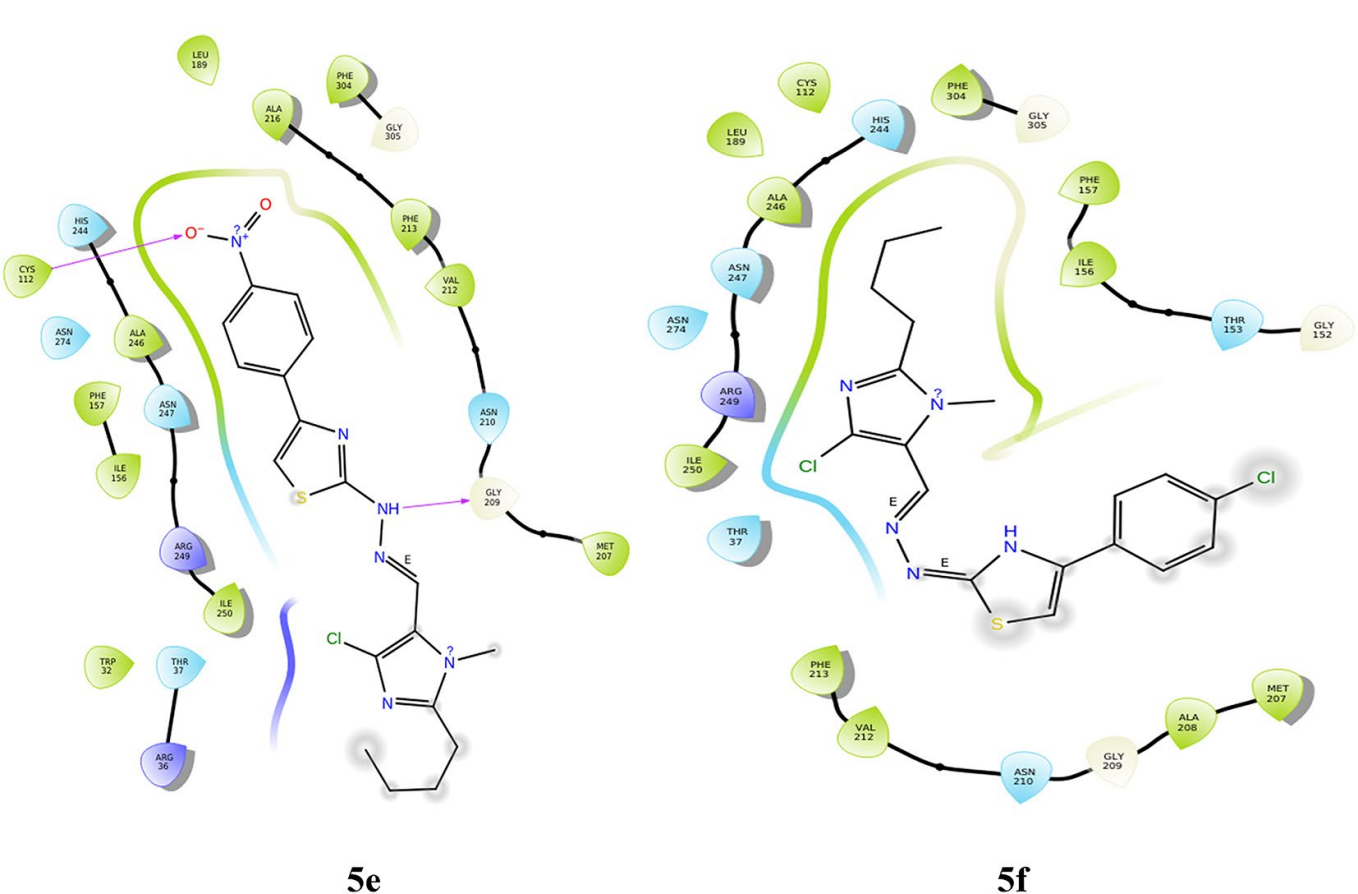
Docking pose of compounds **5a–5f** against 5BNS protein.

The ligand interactions determined through docking revealed intriguing results, with the corresponding poses displayed in Fig. 3. Derivative **5a** shows a п-cation interaction between the imidazole ring and the amino acid **ARG249**. Hydrophobic interactions can be observed around the phenyl ring, and polar interactions towards the thiazole ring. In compound **5b**, the phenyl ring forms п-п stacking interactions with **TRP 32**. The compound also shows hydrophobic and polar interactions as well as good solvent exposure. Derivatives **5a** and **5c** also show п-cation interactions with **ARG 249**. The alkyl chain of imidazole is exposed to the solvent. The hydrogen of the hydroxy group in derivative **5d** bonds with **MET 207** through hydrogen bonding. **CYS 112** forms hydrogen bonds with the nitro group of compound **5e**. The chloro derivative **5f** shows no hydrogen bond or п-stacking interactions, but there are a number of non-bonding interactions. The NH groups of derivatives **5a** and **5e** form hydrogen bonds with **GLY 209**. To validate the docking procedure, the co-crystal structure before docking and the docked co-crystal structure were aligned. The RMSD of the superimposed co-crystal structures before and after docking was found to be 2 Å; however, the bonding interactions with the protein remained unaltered. Table 1 provides a summary of the bonding interactions between the ligand and the protein. Additionally, the RMSD of the superimposed docked ligands and co-crystal structures is within 2 Å for each ligand, further supporting the reliability of the docking results.

**Table 1:** Bonding interactions between ligand (**5a–f**) and 5BNS protein.

Compound	Amino acid	Type of interaction	Site of interaction	RMSD
**5a**	GLY 209	Hydrogen bond	NH of hydrazone	1.51
	ARG 249	п-cation	Imidazole ring	
**5b**	TRP 32	п-п stacking	Benzene ring of OCH_3_ substituent	0.02
**5c**	ARG 249	п-cation	Imidazole ring	0.82
	GLY 209	Hydrogen bond	NH of hydrazone	
**5d**	MET 207	Hydrogen bond	OH substituent	0.02
**5e**	GLY 209	Hydrogen bond	NH of hydrazone	0.02
	CYS 112	Hydrogen bond	Oxygen of NO_2_substituent	
**5f**	–	–	–	0.02

#### Molecular docking with 1EA1

**Fig. 4:**
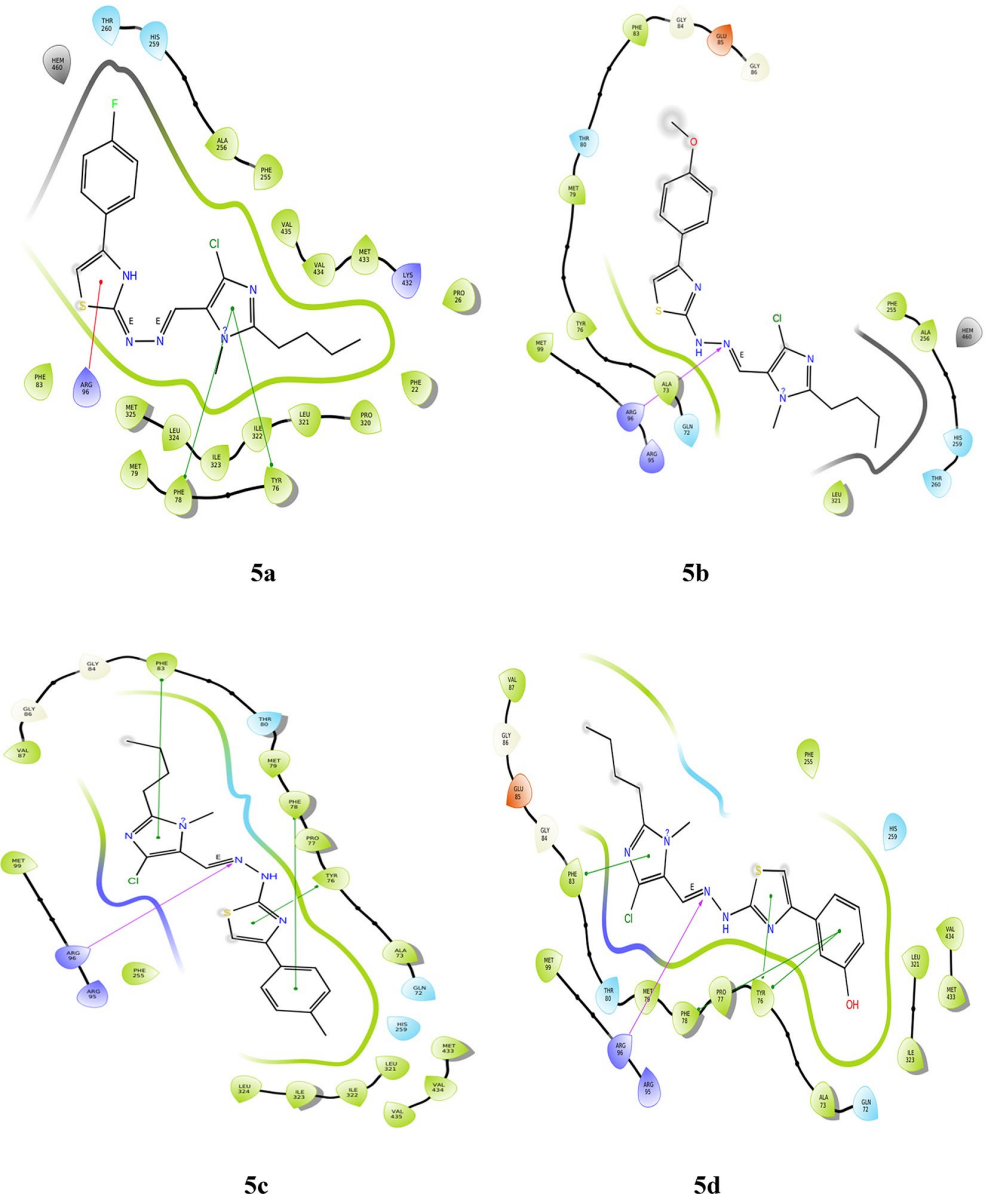

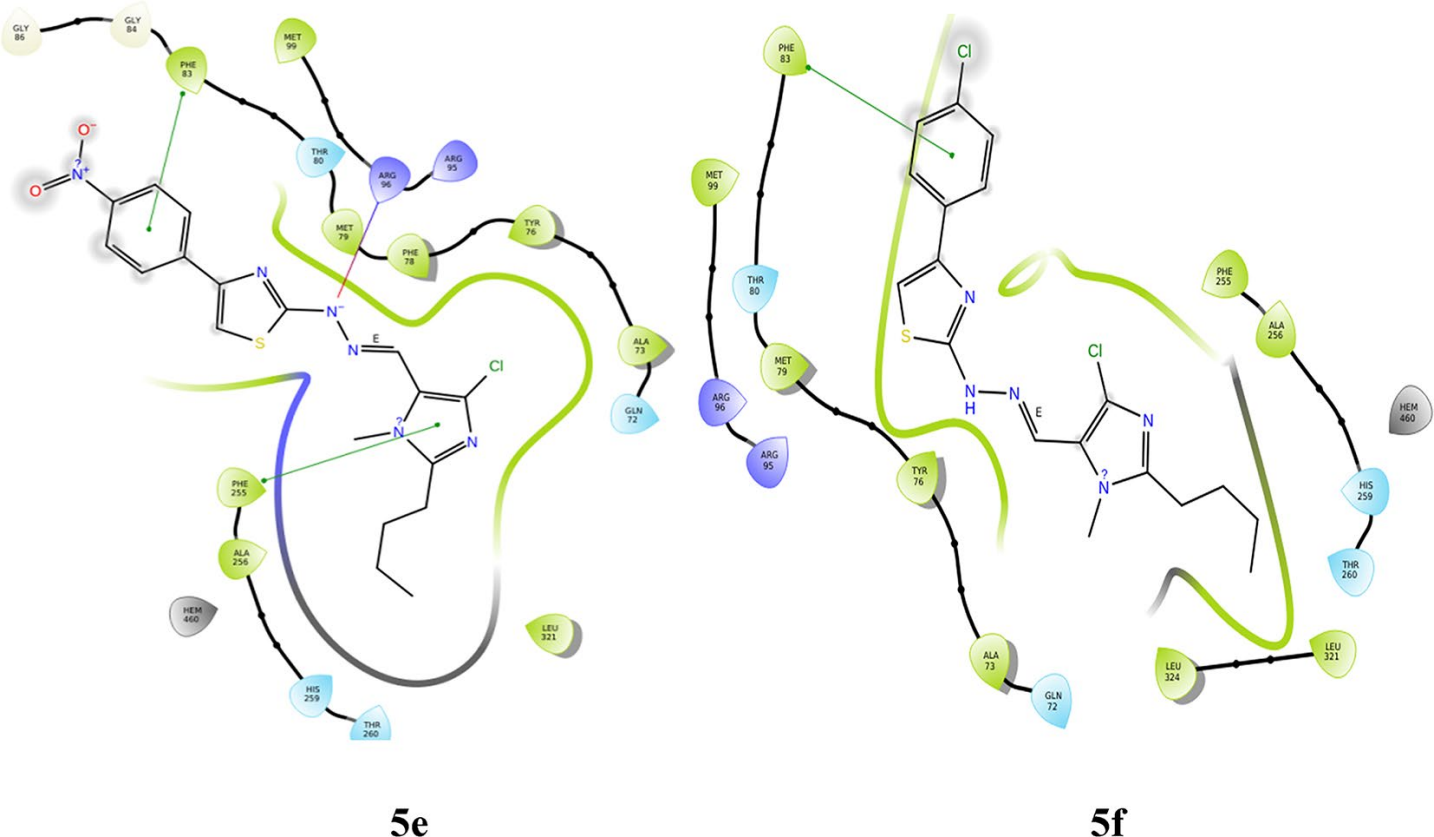
Docking pose of compounds **5a–5f** against 1EA1 protein.

Derivative **5a** displays two п-п stacking interactions between the imidazole ring and **PHE 78** and **TYR 76**. The thiazole ring interacts with **ARG 96** through pi-cation interactions**. ARG 96** forms a hydrogen bond with the imine nitrogen in compound **5b**. In the methyl derivative **5c**, the imidazole ring interacts with the amino acid **PHE 83** via п-п stacking, whereas the thiazole and phenyl rings show the same interaction with **TYR 76** and **PHE 78**, respectively. **ARG 96** interacts with imine nitrogen through hydrogen bonding. The thiazole ring in this derivative has partly charged and partly polar interactions apart from hydrophobic interactions and fits well in the protein active site. In compound **5d**, there are four п-п stacking interactions, one between imidazole and **PHE 83** and one between thiazole and **TYR 76**. The other two compounds are associated with the phenyl ring, which interacts with **TYR 76** and **PHE 78**. Hydrogen bonding between imine nitrogen and **ARG 96** was also observed. The nitro derivative, **5e** shows two п-п stacking interactions with two amino acids, **PHE 83** and **PHE 255**. This derivative has a unique salt bridge between nitrogen and **ARG 96**, which is not found in other derivatives. In derivative **5f**, there is п-п stacking interaction between the phenyl ring and **PHE 83**. The docking poses are represented in Fig. 4. Table 2 represents the summary of bonding interactions between the ligand and the protein. The validation of the docking procedure was carried out, and the RMSD of the superimposed co-crystal structures before and after docking was determined to be 2 Å, with the bonding interactions remaining unaltered. Furthermore, the RMSD of the superimposed docked ligands and co-crystal structures was consistently within 2 Å.

**Table 2:** Bonding interactions between ligand (**5a–f**) and 1EA1 protein.

Compound	Amino acid	Type of interaction	Site of interaction	RMSD
**5a**	ARG 96	п-cation	Thiazole ring	1.51
	PHE 78	п-п stacking	Imidazole ring	
	TYR 76	п-п stacking	Imidazole ring	
**5b**	ARG 96	Hydrogen bonding	Imine nitrogen	0.02
**5c**	PHE 83	п-п stacking	Imidazole ring	1.84
	ARG 96	Hydrogen bond	Imine nitrogen	
	PHE 78	п-п stacking	Benzene ring of CH_3_substituent	
	TYR 76	п-п stacking	Thiazole ring	
**5d**	PHE 83	п-п stacking	Imidazole ring	0.02
	ARG 96	Hydrogen bond	Imine nitrogen	
	TYR 76	п-п stacking	Thiazole ring	
	PHE 78	п-п stacking	Benzene ring of OH substituent	
	TYR 76	п-п stacking	Benzene ring of OH substituent	
**5e**	PHE 83	п-п stacking	Benzene ring of NO_2_ substituent	0.02
	ARG 96	Salt bridge	Nitrogen of hydrazone	
	PHE 255	п-п stacking	Imidazole ring	
**5f**	PHE 83	п-п stacking	Benzene ring of Cl substituent	0.02

#### Molecular docking with 6LUD

**Fig. 5:**
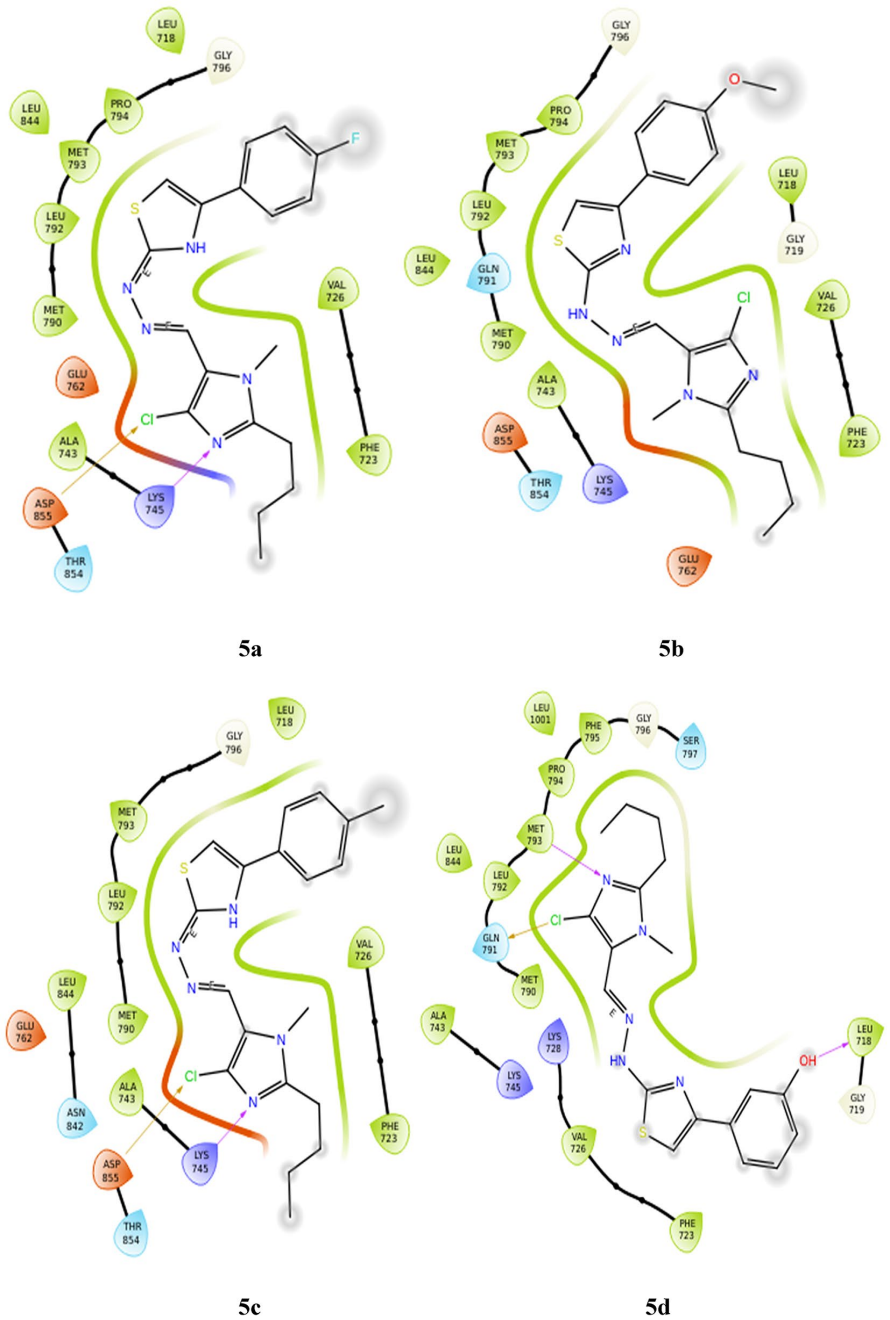

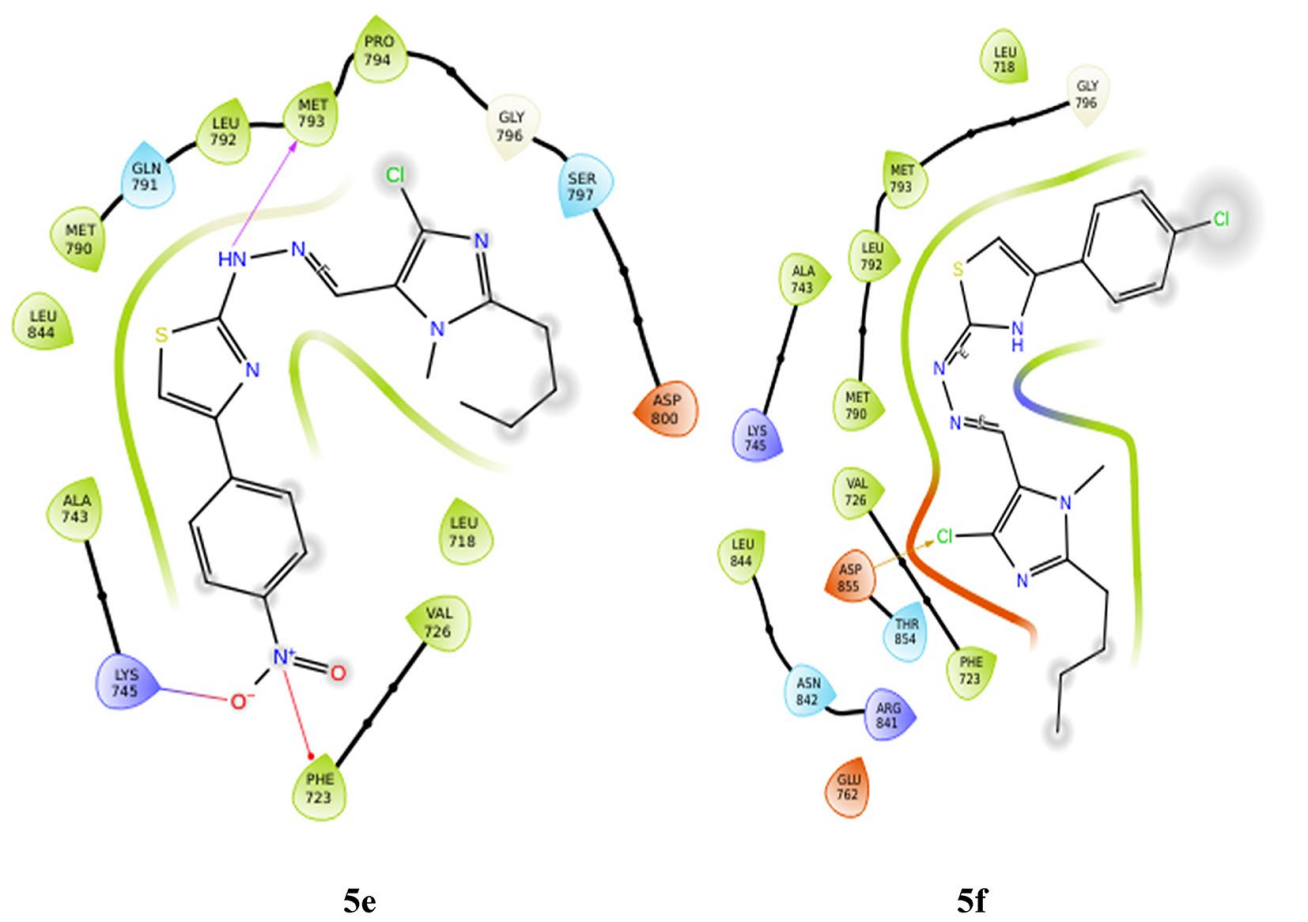
Docking pose of compounds **5a–5f** against 6LUD protein.

**LYS 745** forms hydrogen bonds with the nitrogen of imidazole in two derivatives, **5a** and **5c**. These also show hydrophobic and charged interactions. The methoxy derivative, **5b** displays no bonding interactions but has charged and hydrophobic interactions. All the interactions with the protein are shown in Fig. 5. Compared with the interactions of the other derivatives, the interactions of the hydroxy and nitro derivatives are slightly different. In derivative **5d**, the nitrogen of imidazole forms a hydrogen bond with **MET 793**, and the hydroxy group interacts with **LEU 718** through hydrogen bonding. However, in derivative **5e**, the NH neighboring the imine nitrogen forms a hydrogen bond with **MET 793**, the oxygen from the nitro group forms a salt bridge with **LYS 745**, and the nitrogen of the same substituent interacts with **PHE 723** through a п-cation bond. **5a**, **5c** and **5f** form halogen bonds between the chloro substituent and **ASP 855**, whereas derivative **5d** forms halogen bonds with **GLN 791**. Table 3 gives a summary of the bonding interactions. The docking procedure was validated by calculating the RMSD of the superimposed co-crystal structures before and after docking, which was determined to be 2 Å. This deviation could be due to the rotational bonds in the structure, however the ligand interactions with the amino acids were retained after docking. Similarly, the RMSD of the docked ligands and the co-crystal structures was found to be within 2 Å.

**Table 3:** Bonding interactions between ligand (**5a–f**) and 6LUD protein.

Compound	Amino acid	Type of interaction	Site of interaction	RMSD
**5a**	ASP 855	Halogen bond	Chlroine of imidazole	0.02
	LYS 745	Hydrogen bond	Nitrogen of imidazole	
**5b**	–	–	–	1.53
**5c**	ASP 855	Halogen bond	Chlroine of imidazole	0.01
	LYS 745	Hydrogen bond	Nitrogen of imidazole	
**5d**	MET 793	Hydrogen bond	Nitrogen of imidazole	0.81
	GLN 791	Halogen bond	Chlroine of imidazole	
	LEU 718	Hydrogen bond	OH substituent	
**5e**	MET 793	Hydrogen bond	NH of hydrazone	0.81
	PHE 723	п-cation	Nitrogen of NO_2_substituent	
	LYS 745	Salt bridge	Oxygen of NO_2_ substituent	
**5f**	ASP 855	Halogen bond	Chlroine of imidazole	0.02

#### ADME studies

The ADME properties are crucial for the evaluation of drugs. While the biological and pharmacological activities of the compounds may be influenced by properties related to Lipinski’s rule of five, the rule primarily predicts oral bioavailability. Table 4 shows the parameters of Lipinski’s rule of five. This prediction was performed using the QikProp module, and the results are discussed below.

**Table 4:** Lipinksi’s rule of five parameters.

Compound	Mol. Wt	QPLogPo/W	H-donor	H-acceptor
**5a**	391.89	5.08	1	5.50
**5b**	403.93	5.64	1	4.75
**5c**	387.93	5.22	1	5.50
**5d**	389.90	3.80	2	6.25
**5e**	418.90	4.19	1	6.50
**5f**	408.34	6.08	1	4.00
**RV**	130 to 500	– 2 to 6.5	0 to 6	2 to 20

The molecular weights of the synthesized compounds are well within the range (< 500). There is 1 hydrogen bond donor in all the derivatives except for **5d,** where it is 2 because the hydrogen from OH; and the hydrogen bond acceptors are in the range of 4.00–6.50. The water/gas partition coefficient reveals the hydrophilicity or hydrophobicity of the compounds and is within the expected range. All the synthesized compounds followed Lipinski’s rule of five without violations.

Other parameters listed in Table 5 are equally important for predicting drug properties, such as solubility (QPLogS), percentage oral absorption, blood‒brain barrier permeability (QPLogBB) and polar surface area (PSA). The solubilities of the compounds were measured at 25 °C as a log of their molar concentration; here, compounds **5b**, **5c** and **5f** are outside the recommended range, indicating slightly poor solubility. However, the percentage oral absoption of all the derivatives are very good (100%). Blood–brain barrier permeability provides insight into the diffusion (distribution parameter) of a drug depending upon its binding affinity to blood proteins. Derivative **5f** violates the suggested range and hence is not a BBB permeant. Compared to all the derivatives, **5d** has the lowest QPLogBB value, probably due to the hydroxy substituent. The total solvent accessible surface area (SASA) of the compounds agreed with the range recommended. The Vander Waals surface area of oxygen and nitrogen atoms is represented as the polar surface area, which ranges from 48.58 to 94.07 Å^2^ and is within 200 Å^2^. The ADME data revealed that the synthesized compounds were in good agreement with the recommended values (RV is given in table) for each parameter.

**Table 5:** ADME properties of derivatives **5a–f**

Compound	QPLogS	QPLogBB	%Abs	QPPCaco	SASA	PSA
**5a**	− 6.542	− 0.097	100	3060.34	704.71	49.64
**5b**	− 7.193	− 0.143	100	3299.40	732.27	57.19
**5c**	− 6.958	− 0.204	100	3214.35	738.76	49.86
**5d**	− 5.810	− 1.158	100	507.23	701.33	78.90
**5e**	− 6.459	− 1.343	100	397.29	741.89	94.07
**5f**	− 7.422	− 0.104	100	3279.09	725.10	48.58
**RV**	− 6.5 to 0.5	− 3 to 1.2	Max. 100	< 25 poor > 500 good	300 to 1000	7 to 200

### Antimicrobial analysis

The antimicrobial activity of the derivatives was evaluated by the broth dilution method against three bacterial strains, *E. coli* (Gram-negative), *S. aureus* (Gram-positive), and *M. smegmatis* (Gram-positive), and one fungal strain, *C. albicans*. The test samples were prepared in DMSO (1 mg mL^−1^) and compared with the standards ciprofloxacin (1 mg mL^−1^) and fluconazole (1 mg mL^−1^). The activity results with MIC values (μmol mL^−1^) are given in Table 6. Interestingly, all the derivatives (**5a–f**) displayed appreciable antimicrobial activity. Compounds **5a** and **5f** displayed similar activity against all the bacterial and fungal strains, with MICs of 637.93 μmol mL^−1^ and 306.11 μmol mL^−1^, respectively. This comparable activity of the **5a** and **5f** derivatives could be due to halogen substitution i.e., the presence of fluoro and chloro groups, with the latter showing slightly enhanced activity. Furthermore, compound **5b** showed good activity against *M. smegmatis,* with an MIC of 309.45 μmol mL^−1^. Derivative **5c** exhibited an MIC of 322.89 μmol mL^−1^ against *E. coli* and *M. smegmatis* but 645.78 μmol mL^−1^ against *S. aureus* and *C. albicans.* The MIC values differ from bacteria to bacteria, although they belong to Gram-positive strains; nevertheless, the compounds are active against both strains. Derivative **5d** with a hydroxy (OH) group was fairly active against *E. coli* bearing a MIC of 320.50 μmol mL^−1^. The compound with a nitro substituent, **5e** (298.40 μmol mL^−1^), exhibited better antifungal activity against *C. albicans* compared to the other bacterial strains.

**Table 6:** Antimicrobial activity of derivatives **5a–f** (MICs are given in μmol mL^−1^).

Compounds	*E. coli*	*S. aureus*	*M. smegmatis*	*C. albicans*
**5a**	637.93	637.93	637.93	637.93
**5b**	618.92	618.92	309.45	618.92
**5c**	645.78	322.89	322.89	645.78
**5d**	641.19	320.50	641.19	641.19
**5e**	298.40	596.80	596.80	298.40
**5f**	306.11	306.11	306.11	306.11
**Ciprofloxacin**	23.54	23.54	23.54	–
**Fluconazole**	–	–	–	25.47

### In vitro cytotoxicity studies

**Fig. 6:**
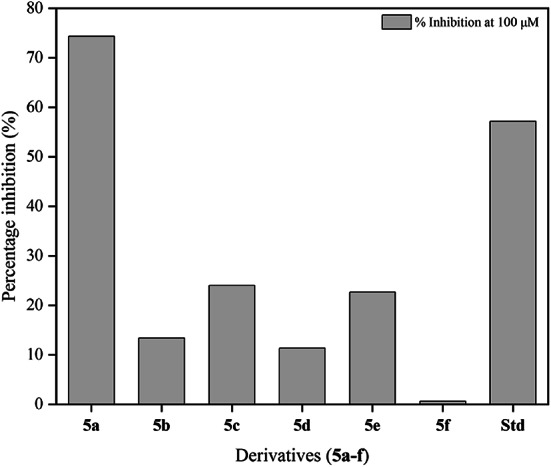
Bar graph representing the percentage inhibition of derivatives **5a-f** at a 100 μM concentration.

**Fig. 7:**
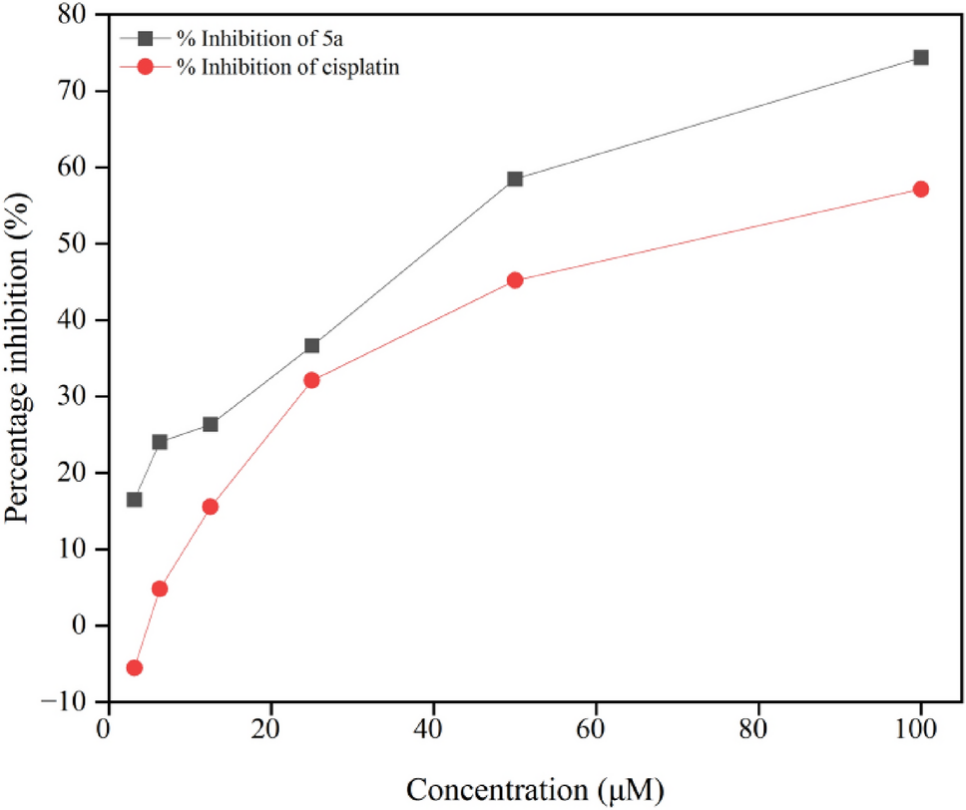
Percentage inhibition of derivative **5a** and cisplatin at 100 μM concentration.

Cytotoxicity studies of the derivatives were performed via SRB assay. All six compounds with cisplatin as the standard were evaluated. Among these derivatives, fluoro-substituted **5a** displayed excellent activity with an IC_50_ of 33.52 μM compared to the standard, which showed an IC_50_ of 67.67 μM. The IC_50_ of the other derivatives **5b-f** were greater than 100 μM. The percentage inhibition of all the derivatives **5a-f** and the standard, cisplatin at 100 μM is given in Table 7. Figure 6 shows that derivatives **5b** (methoxy substituent at the fourth position) and **5d** (hydroxy substituent at the third position) exhibit similar activity against the MCF-7 cell line, with a percentage inhibition of approximately 11–13%. About 22–24% inhibition is seen for the methyl and nitro derivatives, **5c** and **5e**, respectively. However, the standard showed about 57% inhibition at a concentration of 100 μM, which is lower than that of derivative **5a**. Derivative **5f** showed negligible inhibition at the same concentration, indicating that it is inactive against the cell line. Figure 7 shows the percentage inhibition of derivative **5a** and cisplatin against the MCF-7 cell line at different concentrations (3.125 μM, 6.25 μM, 12.5 μM, 25 μM, 50 μM and 100 μM).

**Table 7:** Percentage inhibition of derivatives **5a-f** at a concentration of 100 μM.

Derivatives	Percentage inhibition (%) at 100 μM concentration
**5a**	74.33
**5b**	13.36
**5c**	24.02
**5d**	11.33
**5e**	22.64
**5f**	0.60
**Cisplatin (Std)**	57.12

### Molecular dynamics (MD) simulation

**Fig. 8:**
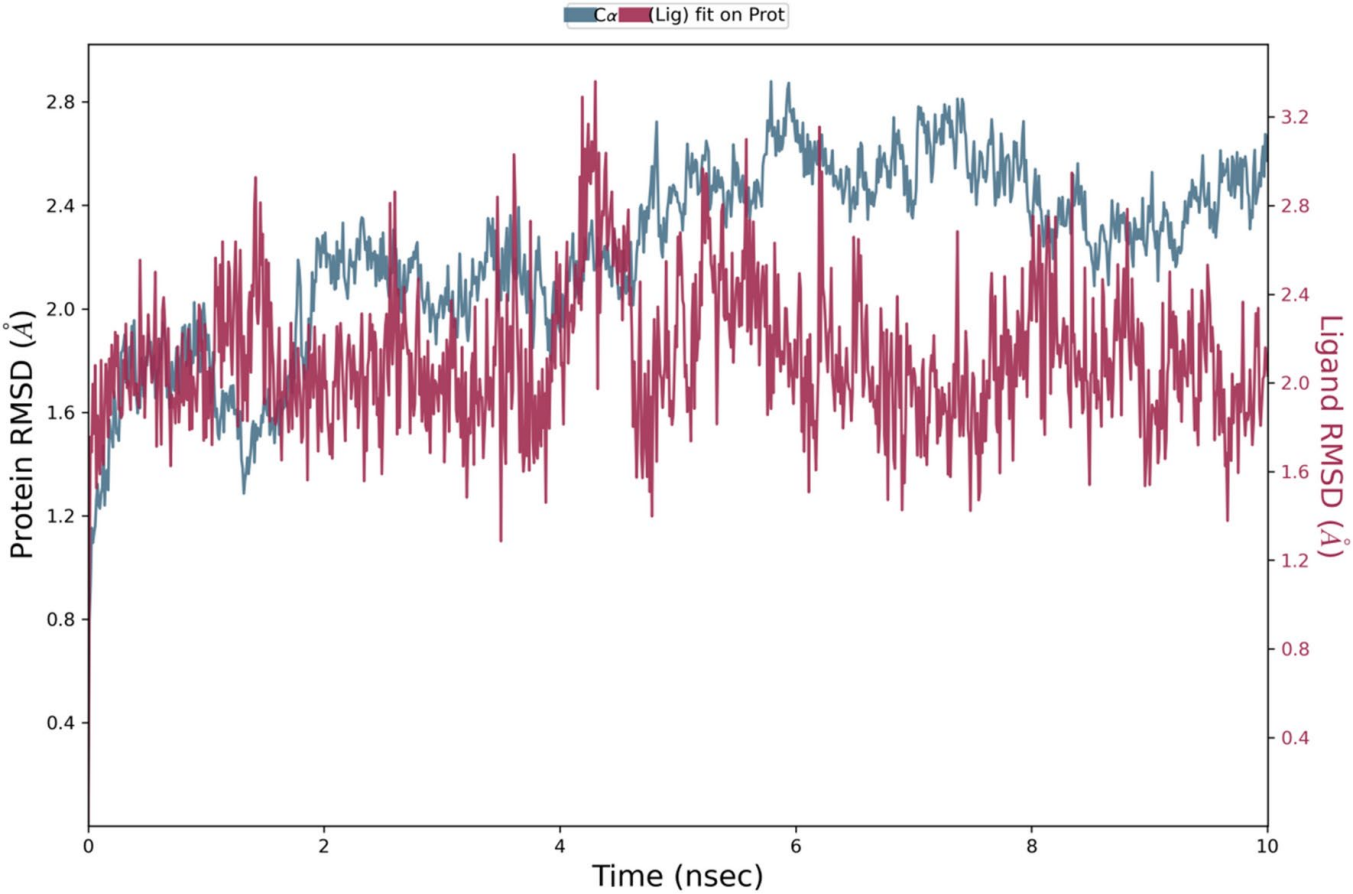
RMSD plot of the protein‒ligand complex (EGFR-5a) during the simulation.

**Fig. 9:**
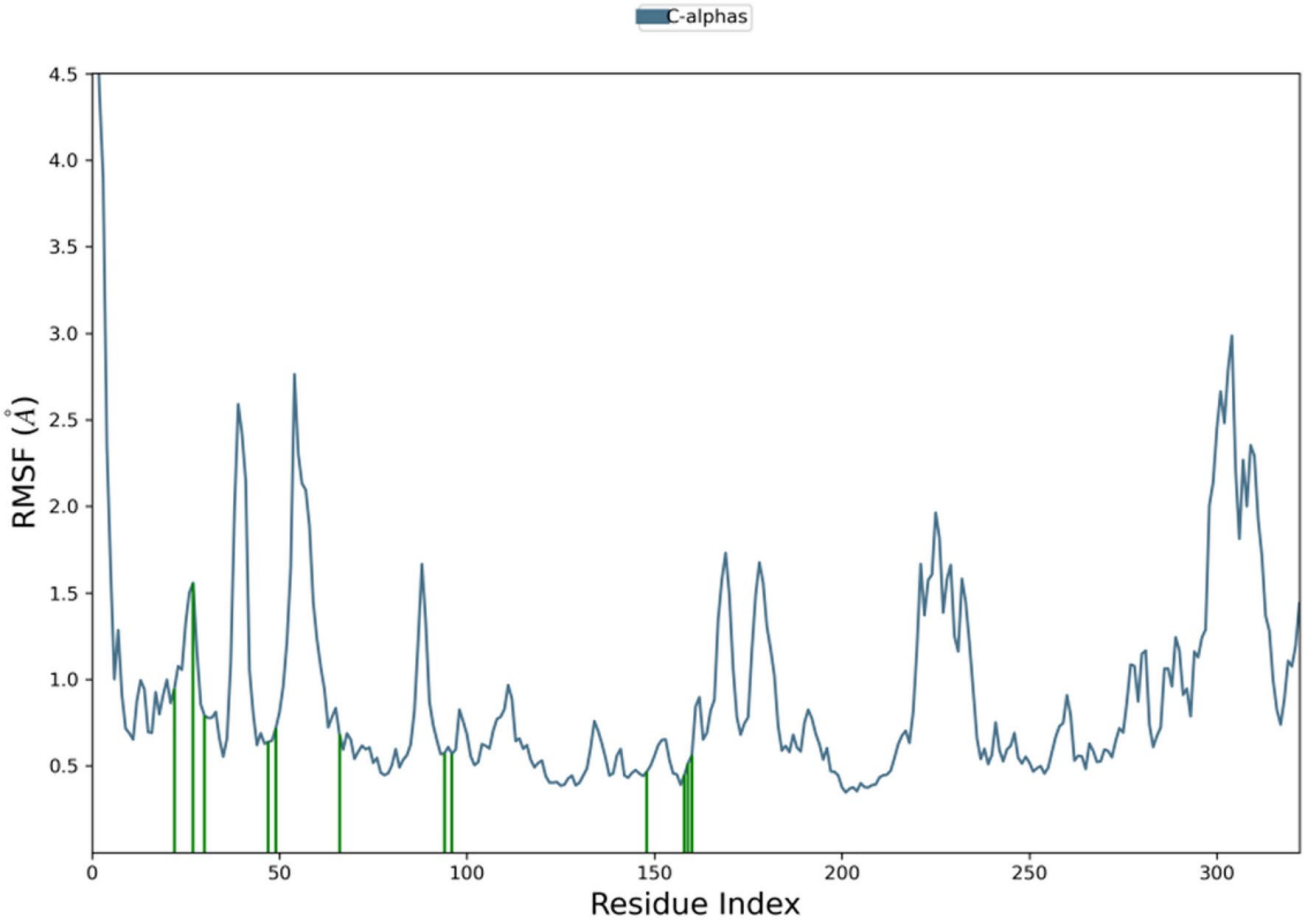
RMSF map of the protein–ligand complex (EGFR-5a) during the simulation.

**Fig. 10:**
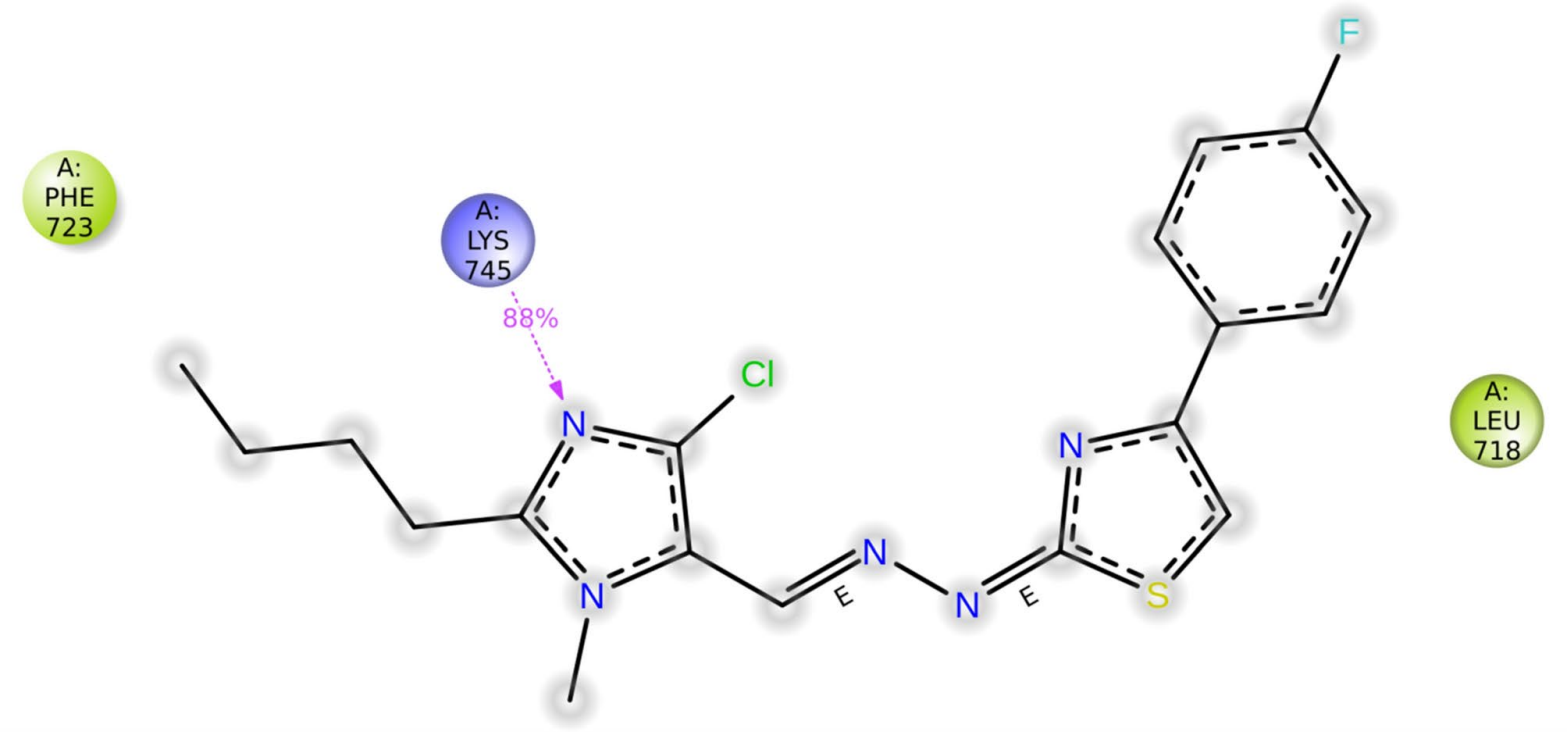
Ligand interactions with protein residues throughout the trajectory.

**Fig. 11:**
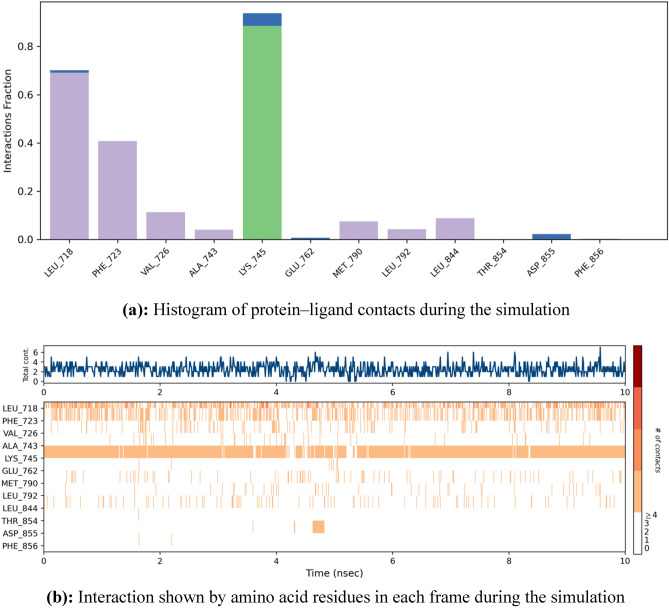
(**a**) Histogram of protein‒ligand contacts during the simulation. (**b**) Interaction shown by amino acid residues in each frame during the simulation.

As the derivative **5a** exhibited excellent cytotoxicity, it’s stability within the active site of the protein 6LUD was evaluated by MD simulations. The root mean square deviation (RMSD) for the protein–ligand complex (EGFR-5a) was in equilibrium upto 5 ns post which some fluctuations were observed upto 8 ns, as represented in Fig. 8. The deviation was less than 2 Å, which is well within the acceptable range for small proteins indicating that the complex was stable during the simulation. The root mean square fluctuation (RMSF) shown in Fig. 9 represents the changes that take place locally along the protein chain. Comparing the XP-ligand docking interaction of **5a** and MD ligand docking interaction, the ligand retains the hydrogen bond with **LYS 745**. The protein ligand contacts during the simulation helps in understanding the type of interactions being made. Here, hydrogen bond of **LYS 745** can be observed more than 80% of the simulation time (Fig. 10) and **LEU 718** residue is estimated to be involved in hydrophobic interaction for about 70% of the simulation as shown in Fig. 11a. Interaction shown by amino acid residues in each frame during the simulation is represented in Fig. 11b. This gives an insight into the satisfactory stability of the complex.

### Structure–activity relationship (SAR) studies

The SAR with respect to antimicrobial activity revealed that the derivatives showed similar activity against Gram-positive and Gram–negative bacteria. Compound **5e**, bearing a nitro group at the fourth position, and compound **5f**, with a chloro substituent at the same position, exhibited comparable efficacy against *E. coli*, a Gram-negavtive bacterium, with an MIC of 298.40 μmol mL^−1^ and 306.11 μmol mL^−1^, respectively. Notably, both derivatives **5e** and **5f** have electron-withdrawing groups. In the case of Gram-positive strains *S. aureus* and *M. smegmatis*, derivatives **5c** and **5f** exhibited analogous activity, despite the former bearing an electron-donating substituent, a methyl group and the latter having an electron-withdrawing substituent. This suggests that the structure of the derivatives plays a more crucial role in the activity rather than just their electron donating or withdrawing abilities. However, derivatives **5b** and **5d**, bearing the electron-donating substituents methoxy and hydroxy, respectively, showed similar activities against *M. smegmatis* and *E. coli*. Derivative **5a** with fluoro substituent had an MIC of 637.93 μmol mL^−1^ against all the bacterial and fungal strains. Derivatives **5e** and **5f** also displayed good activity against *C. albicans*, a fungal strain. However, in vitro cytotoxicity studies revealed that the fluoro derivative at the fourth position, **5a**, is the most active against the MCF-7 cell line. Derivatives with methoxy (**5b**) and hydroxy (**5d**) groups, i.e., electron donating groups at the fourth position, displayed similar inhibition; likewise, derivatives **5c** and **5e** exhibited inhibition of approximately the same percentage. Therefore, all the derivatives **5a-f** have shown satisfactory activity against all microbes, and derivative **5a** displays better cytotoxicity against MCF-7 cells, with electron withdrawing substituents having a dominant effect on both in vitro activities.

## Experimental section

### Chemistry

The reactants that have been used in the synthesis are procured from Spectrochem. The reagents and solvents were procured from commercial sources and used without further purification. Thin layer chromatography (TLC) was conducted on 0.25 mm silica gel plates purchased from Merck. The TLC plate was analyzed using ultraviolet light (UV R-340). The melting points of the compounds were estimated using a Thiel’s tube. The IR spectra of all the compounds were recorded using a Shimadzu FTIR instrument. ^1^H NMR and ^13^C NMR spectra of all the compounds were recorded on a Bruker AM 400 MHz and 100 MHz NMR spectrometer using DMSO as the solvent and TMS as the internal standard. The mass spectra of the derivatives were determined using mass spectrometry-LC‒MS/MS; model: Synapt G2 high detection mass spectrometer.

#### General method for the synthesis of 2-butyl-4-chloro-1-methyl-1H-imidazole-5-carbaldehyde (2)

An equimolar ratio of 2-butyl-4-chloro-1H-imidazole-5-carbaldehyde (**1)** and methyl iodide was reacted in DMF in the presence of base K_2_CO_3_ for three hours at 85 °C. The progress of the reaction was monitored by TLC. After completion, water was added to the reaction mixture, and the mixture was extracted twice with ethyl acetate. The organic layer was dried over anhydrous sodium sulfate and evaporated to obtain oily product **2** (90%yield).

#### General method for the synthesis of 2-((2-butyl-4-chloro-1-methyl-1H-imidazol-5-yl)methylene)hydrazinecarbothioamide (3)

Compound **2** and thiosemicarbazide were refluxed at equimolar ratios for three hours in ethanol. After the completion of the reaction, it was allowed to cool, and the solid was filtered with cold water. The obtained yield was 85%.

#### General method for the synthesis of 2-(2-((2-butyl-4-chloro-1-methyl-1H-imidazol-5-yl)methylene)hydrazinyl)-4-phenylthiazole derivatives 5a-f

Equimolar ratios of **3** and various substituted phenacyl bromides (**4a-f**) were condensed for 30 min, and the reaction progress was checked using TLC. After the completion of the reaction, the mixture was allowed to cool, and the formed solid was filtered and recrystallized using methanol.

*2-(2-((2-butyl-4-chloro-1-methyl-1H-imidazol-5-yl)methylene)hydrazinyl)-4-(4-fluorophenyl)thiazole* (**5a**). 80% yield. M.p.: 196–198 °C.IR: ν_max_: 1628 (C=N), 1485 (Ar C=C), 1273 (C-N), 791 (C–Cl) cm^−1^; ^1^H-NMR (DMSO*,* 400 MHz) δ, ppm (*J*, Hz): 8.00 (1H, s, ArH); 7.91 (2H, m, ArH); 7.32 (1H, s, ArH); 7.27 (2H, t, *J* = 8, ArH); 5.68 (1H, s, NH); 3.84 (3H, s, CH_3_); 2.72 (2H, t, *J* = 12, CH_2_); 1.66 (2H, m, CH_2_); 1.40 (2H, m, CH_2_); 0.94 (3H, t, *J* = 12, CH_3_).^13^C-NMR (100 MHz, DMSO) δ (ppm): 168.5, 163.3, 160.9, 150.7, 149.5, 131.4, 130.9, 128.4, 127.9, 121.8, 116.0, 115.8, 103.9, 33.6, 29.0, 25.9, 22.1, 14.1. TOF–MS (m/z) = 392.19(M + H)^+^; Retention time—5.05.

*2-(2-((2-butyl-4-chloro-1-methyl-1H-imidazol-5-yl)methylene)hydrazinyl)-4-(4-methoxyphenyl)thiazole* (**5b**). 85% yield. M.p.: 200–202 °C. IR: ν_max_: 2964 (aliphatic CH), 1611 (C=N), 1487 (Ar C=C), 1270 (C-N), 1249 (C-O), 775 (C–Cl) cm^−1^; ^1^H-NMR (DMSO*,* 400 MHz) δ, ppm (*J*, Hz): 7.99 (1H, s, ArH); 7.79 (2H, d, *J* = 8, ArH); 7.16 (1H, s, CH), 6.99 (2H, d, *J* = 8, ArH); 3.84 (3H, s, CH_3_); 3.79 (3H, s, CH_3_); 2.72 (2H, t, *J* = 12, CH_2_); 1.66 (2H, m, CH_2_); 1.40 (2H, m, CH_2_); 0.94 (3H, t, *J* = 12, CH_3_). ^13^C-NMR (100 MHz, DMSO) δ (ppm): 168.4, 159.4, 150.8, 149.9, 131.3, 128.9, 127.4, 127.3, 121.7, 114.4, 102.0, 55.6, 33.5, 29.0, 25.9, 22.1, 14.1. TOF–MS (m/z) = 404.21(M + H)^+^; Retention time—4.92.

*2-(2-((2-butyl-4-chloro-1-methyl-1H-imidazol-5-yl)methylene)hydrazinyl)-4-(p-tolyl)thiazole* (**5c**). 80% yield. M.p.: 195–197 °C.IR: ν_max_: 3318 (NH), 1619 (C=N), 1485 (Ar C=C), 1269 (C-N), 770 (C–Cl) cm^−1^; ^1^H-NMR (DMSO*,* 400 MHz) δ, ppm (*J*, Hz): 8.00 (1H, s, ArH); 7.75 (2H, d, *J* = 8, ArH); 7.25 (2H, d, *J* = 8, ArH), 7.21 (1H, s, CH); 3.84 (3H, s, CH_3_); 2.72 (2H, t, *J* = 12, CH_2_); 2.33 (3H, s, CH_3_); 1.66 (2H, m, CH_2_); 1.40 (2H, m, CH_2_); 0.94 (3H, t, *J* = 12, CH_3_). ^13^C-NMR (100 MHz, DMSO) δ (ppm): 168.4, 150.8, 150.4, 137.5, 131.9, 131.2, 129.6, 129.0, 125.9, 121.7, 103.2, 33.5, 29.0, 25.9, 22.1, 21.2, 14.1. TOF–MS (m/z) = 388.22(M + H)^+^; Retention time—5.28.

*3-(2-(2-((2-butyl-4-chloro-1-methyl-1H-imidazol-5-yl)methylene)hydrazinyl)thiazol-4-yl)phenol* (**5d**). 68% yield. M.p.: 211–213 °C.IR: ν_max_: 3389 (OH), 1621 (C = N), 1485 (Ar C=C), 1276 (C-N), 738 (C–Cl) cm^−1^; ^1^H-NMR (DMSO*,* 400 MHz) δ, ppm (*J*, Hz): 8.00 (1H, s, ArH), 7.28 (4H, m, ArH), 6.72 (1H, d, *J* = 8, ArH); 6.00 (2H, s, OH, NH); 3.84 (3H, s, CH_3_); 2.72 (2H, t, *J* = 12, CH_2_); 1.66 (2H, m, CH_2_); 1.40 (2H, m, CH_2_); 0.94 (3H, t, *J* = 12, CH_3_). ^13^C-NMR (100 MHz, DMSO) δ (ppm): 168.3, 158.0, 150.8, 150.6, 135.9, 131.1, 130.0, 129.0, 121.7, 116.8, 115.2, 113.0, 104.0, 33.5, 29.0, 26.0, 22.1, 14.1.

*2-(2-((2-butyl-4-chloro-1-methyl-1H-imidazol-5-yl)methylene)hydrazinyl)-4-(4-nitrophenyl)thiazole* (**5e**). 85% yield. M.p.: 209–211 °C. IR: ν_max_: 2964 (aliphatic CH), 1580 (C=N), 1515 (N–O), 1444 (Ar C=C), 1272 (C-N), 718 (C–Cl) cm^−1^; ^1^H-NMR (DMSO*,* 400 MHz) δ, ppm (*J*, Hz): 12.25 (1H, s, NH), 8.28 (2H, d, *J* = 8, ArH), 8.11 (2H, d, *J* = 8.8, ArH), 7.99 (1H, s, ArH), 7.72 (1H, s, CH), 3.82 (3H, s, CH_3_), 2.70 (2H, t, *J* = 12, CH_2_), 1.66 (2H, m, CH_2_), 1.40 (2H, m, CH_2_), 0.93 (3H, t,*J* = 12, CH_3_). ^13^C-NMR (100 MHz, DMSO) δ (ppm): 168.8, 151.0, 149.0, 146.6, 141.0, 131.5, 130.1, 126.8, 124.5, 121.4, 108.9, 33.4, 29.1, 26.1, 22.2, 14.1.TOF–MS (m/z) = 419.19(M + H)^+^; Retention time—5.03.

*2-(2-((2-butyl-4-chloro-1-methyl-1H-imidazol-5-yl)methylene)hydrazinyl)-4-(4-chlorophenyl)thiazole* (**5f**). 80% yield. M.p.: 200–202 °C. IR: ν_max_: 2957 (aliphatic CH), 1627 (C=N), 1490 (Ar C=C), 1270 (C-N), 772 (C–Cl) cm^−1^; ^1^H-NMR (DMSO*,* 400 MHz) δ, ppm (*J*, Hz): 7.99 (1H, s, ArH); 7.86 (2H, m, ArH); 7.46 (2H, m, ArH); 7.39 (1H, s, ArH); 3.83 (3H, s, CH_3_); 2.69 (2H, t, *J* = 12, CH_2_); 1.63 (2H, m, CH_2_); 1.36 (2H, m, CH_2_); 0.92 (3H, t, *J* = 12, CH_3_). ^13^C-NMR (100 MHz, DMSO) δ (ppm): 168.6, 150.9, 149.7, 133.8, 132.4, 131.2, 129.6, 129.1, 127.7, 121.5, 104.9, 33.4, 29.1, 26.0, 22.1, 14.1. TOF–MS (m/z) = 410.17(M + 2)^+^; Retention time—5.43.

### Molecular docking

In-silico studies were accomplished using Schrodinger software to determine the interactions between the protein and ligand through docking. New series of compounds were designed and evaluated for antimicrobial activity. Initially, 2D sketcher was used to construct standards and ligands, and then the LigPrep tool was used for ligand preparation. The protein was chosen from the RCSB protein data bank and imported. The protein was further optimized and minimized after removing the solvents using a protein preparation wizard. The prepared protein was docked with the prepared ligands after generating a grid. The Qikprop tool was used to analyze the ADME (absorption, distribution, metabolism and excretion) properties of the developed compounds. For antibacterial and antifungal molecular docking, *E. coli* Fabh with small molecule inhibitor 2 (PDB: 5BNS) with a resolution of 2.20 Å and Cytochrome P450 14 alpha-sterol demethylase (CYP51) from *Mycobacterium tuberculosis* in complex with fluconazole (PDB: 1EA1) with a resolution of 2.21 Å; and crystal structure of EGFR in complex with osimertinib (PDB: 6LUD) were chosen from the Protein Data Bank.

### Antimicrobial activity

The antimicrobial activity of the synthesized derivatives was evaluated by the microdilution method, and the minimum inhibitory concentration (MIC), i.e., the lowest concentration at which the drug obstructs microbial growth, was determined. The compounds were tested against Gram-positive bacteria *Staphylococcus aureus* MTCC 3160 and *Mycobacterium smegmatis* MTCC 944; Gram-negative bacteria *Escherichia coli* MTCC 1687 and *Candida albicans* MTCC 7523. All the samples were prepared in DMSO at a concentration of 1 mg mL^−1^. Then 100 μL of this sample (1 mg mL^−1^) was added to the top well (1A) of a 96-well plate and serially diluted until reaching the well 1H. This process of dilution was repeated for all the samples top to bottom of the plate (A-H) (100–0.78 μL). This was followed by the addition of 50 μL of nutrient broth and 50 μL of diluted bacterial or fungal culture (100 times diluted 0.5 McFarland culture). The control microbial cultures were processed in triplicate in a manner similar to that used for the samples without any inhibitors*,* and 100% growth was considered as the negative control. The antibacterial standard (ciprofloxacin) and antifungal standard (fluconazole) were also diluted serially from 100 to 0.78 μL from wells A-H, similar to the samples. All the cultures in the 96*-*well plates were incubated for 12–24 h at room temperature, and the OD was read at 600 nm. Ten micro liters of resazurin dye (0.001%) was added to each well of the 96-well plates, and the color change was checked after 15–30 min to confirm the antimicrobial activity and the MIC^23^.

### In vitro cytotoxicity studies

The synthesized derivatives were evaluated for their cytotoxicity against the MCF-7 cell line (NCCS, Pune) by Sulforhodamine B (SRB) assay. Human breast cancer cells (MCF-7) were cultured in Dulbecco’s Modified Eagle’s Medium (DMEM) (Sigma Aldrich, India) supplemented with 10% fetal bovine serum (FBS) (Invitrogen) and 1% penicillin/streptomycin (Sigma Aldrich, India) and incubated in a humidified incubator with 5% CO_2_ at 37 °C. The SRB assay was performed using the method described by Vichai et al. MCF-7 cells were seeded at a density of 5000 cells per well in a 96-well plate and incubated for 48 h at 37 °C in an incubator with a 5% CO_2_ supply. After monolayer formation, the cells were treated with different concentrations of cisplatin (standard drug) and the test compounds and incubated for 48 h. The cells were then fixed by incubation with ice-cold 10% trichloroacetic acid (TCA) (SRL) for one hour at 4 °C. After cell fixation, the cells were washed with water three times to remove excess TCA, followed by air drying of the plates. The dried plates were stained with SRB dye (Sigma Aldrich, India) for 30 min in the dark and then rinsed with 1% acetic acid (Sigma Aldrich, India) to remove any additional dye. The bound dye was then dissolved in 10 mM Tris base (Himedia), and the absorbance was measured at 530 nm^24^.

### Molecular dynamics (MD) simulation

The ligand **5a** was docked to the anticancer target EGFR (PDB: 6LUD) and MD simulation was carried out for EGFR-5a complex. The system builder module of Desmond was used to build the system where the protein–ligand complex was solvated in an orthorhombic box containing a TIP3P solvent system. The box’s volume was 10 × 10 × 10 Å, and the built system was minimized to attain the equilibration. 0.15 M NaCl salt concentrations were added and the system was auto-neutralised by recalculating the required ions and was run with an OPLS4 force field. The simulation was performed using the molecular dynamics module by loading from the workspace for a period of 10 ns. The model system was relaxed before simulation with normal pressure temperature (NPT) at 1.01325 bar pressure and 300 K temperature using the Martina-Tobias-Klein method as barostat and Norse-Hoover chain method as thermostat respectively^25^.

## Conclusion

In conclusion, a series of six novel compounds were successfully synthesized in three synthetic steps and comprehensively characterized using infrared (IR) spectroscopy, nuclear magnetic resonance (NMR), and mass spectrometry. The biological evaluation of these compounds against three bacterial strains and a fungal strain demonstrated promising antimicrobial activity, with the imidazole and thiazole scaffolds identified as key structural elements contributing to their efficacy. Notably, derivative **5f** exhibited the lowest minimum inhibitory concentration (MIC) across all tested strains, while derivative **5a** displayed significant anticancer activity, with an IC_50_ of 33.52 μM in the MCF-7 cytotoxicity assay, outperforming the standard chemotherapeutic agent cisplatin, with an IC_50_ of 67.67 μM. Molecular docking studies further elucidated the binding interactions between the compounds and their respective target proteins, while molecular dynamics simulations of derivative **5a** within the active site of protein 6LUD indicated remarkable stability throughout the simulation period. These findings suggest that derivative **5a** warrants further exploration for its potential in therapeutic applications, particularly in cancer treatment.

## Supplementary Information

Supplementary Information.

## Data Availability

Data is provided within the manuscript or supplementary information files.
