# Supplementary material for: Synthesis, characterization and in silico studies of novel multifunctional imidazole-thiazole hybrids with potent antimicrobial and anticancer properties
Source: Sci Rep. 2025 Mar 21;15:9809. doi: 10.1038/s41598-025-93249-1 (PMC11928448; doi:10.1038/s41598-025-93249-1)
Supplement: Supplementary file 1 — Supplementary Information. [file 41598_2025_93249_MOESM1_ESM.docx]

**Synthesis, Characterization and In Silico studies of Novel Multifunctional Imidazole-Thiazole Hybrids with Potent Antimicrobial and Anticancer Properties**

**FTIR data of derivatives 5a-5f**


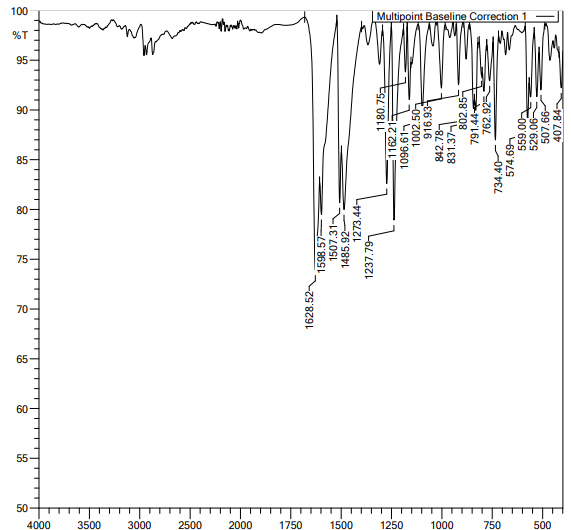


**Figure S1:** IR spectrum of compound **5a**


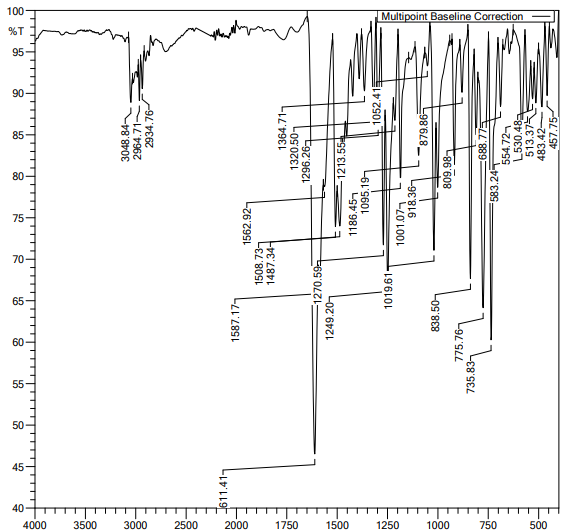


**Figure S2:** IR spectrum of compound **5b**


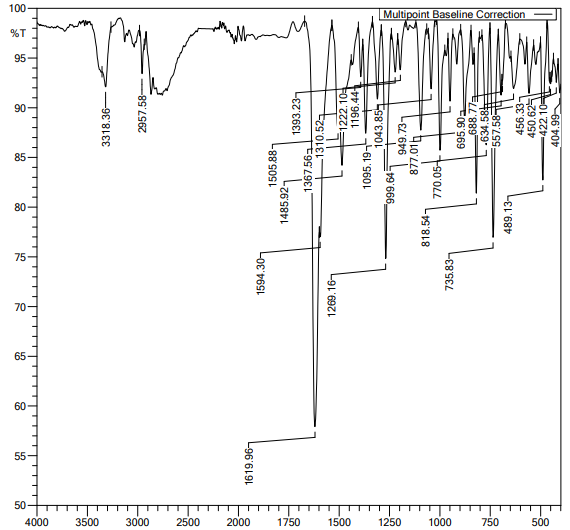


**Figure S3:** IR spectrum of compound **5c**


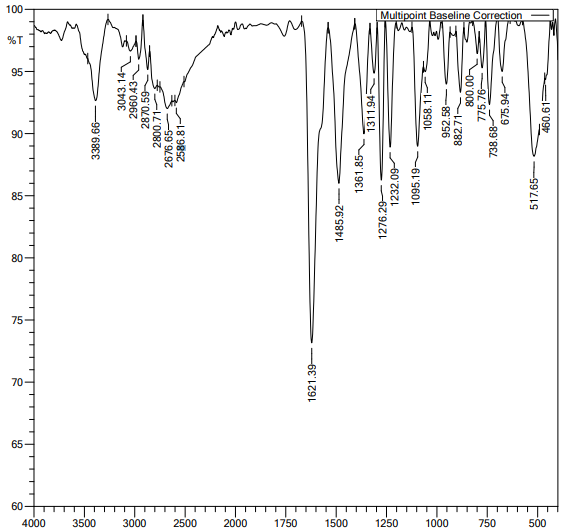


**Figure S4:** IR spectrum of compound **5d**

**
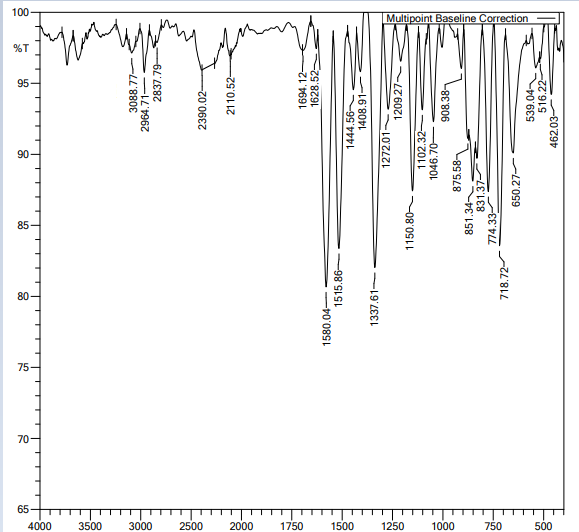
**

**Figure S5:** IR spectrum of compound **5e**

**
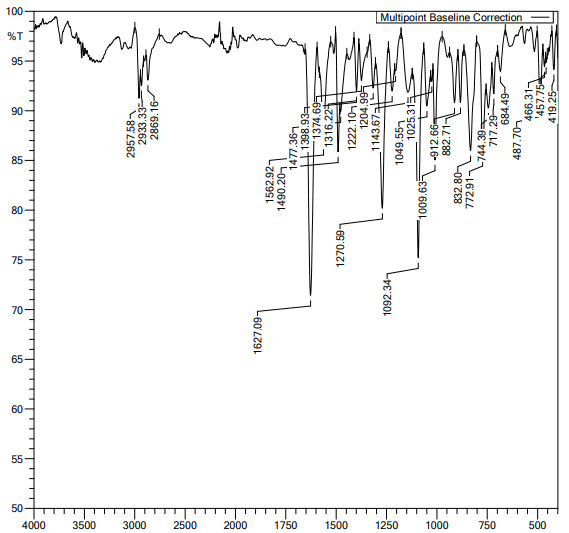
**

**Figure S6:** IR spectrum of compound **5f**

**^1^HNMR data of derivatives 5a-5f**


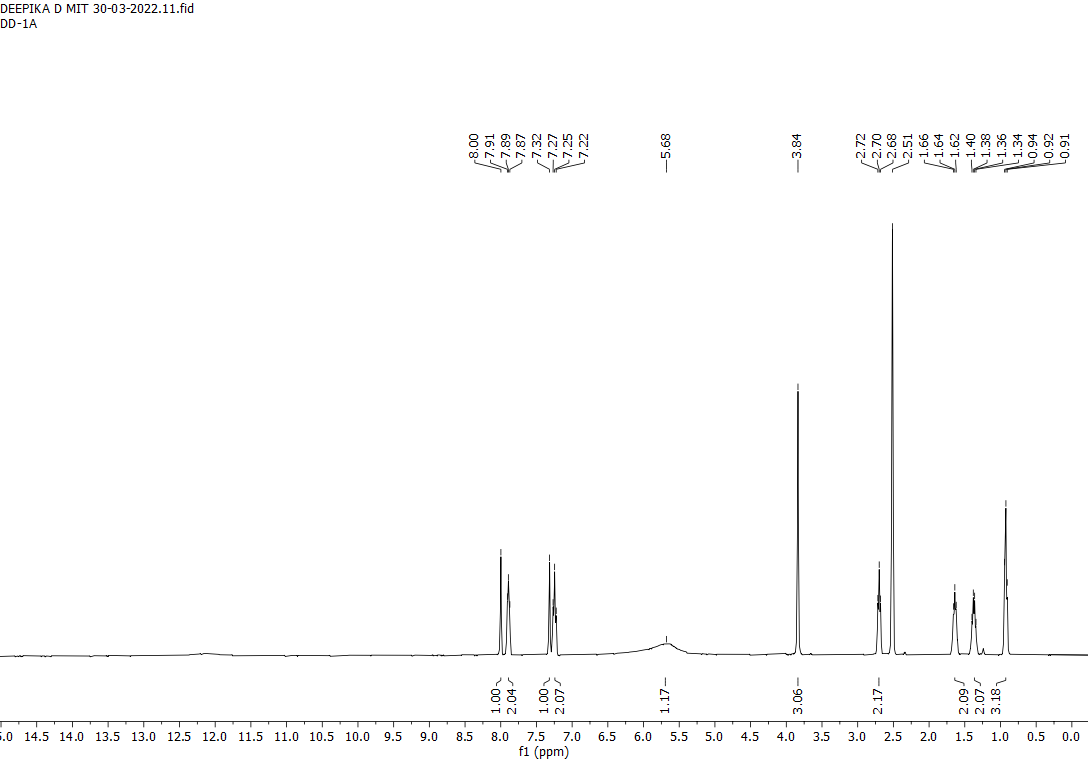


**Figure S7:** ^1^H NMR spectrum of **5a**


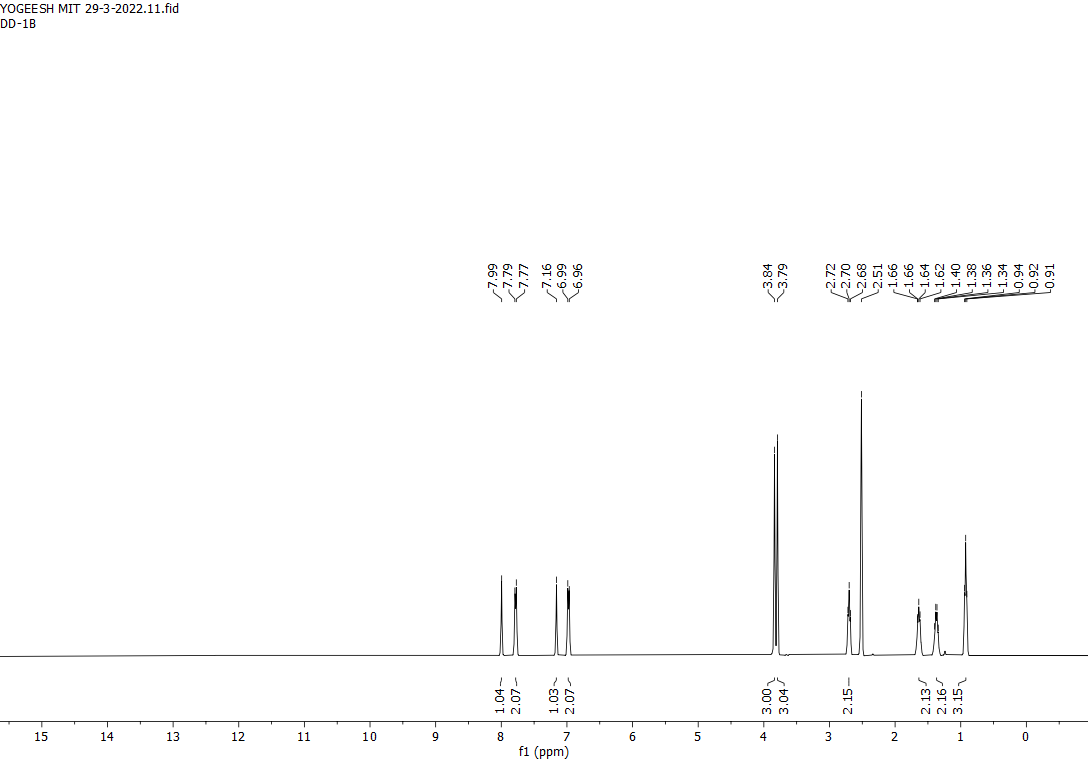


**Figure S8:** ^1^H NMR spectrum of **5b**


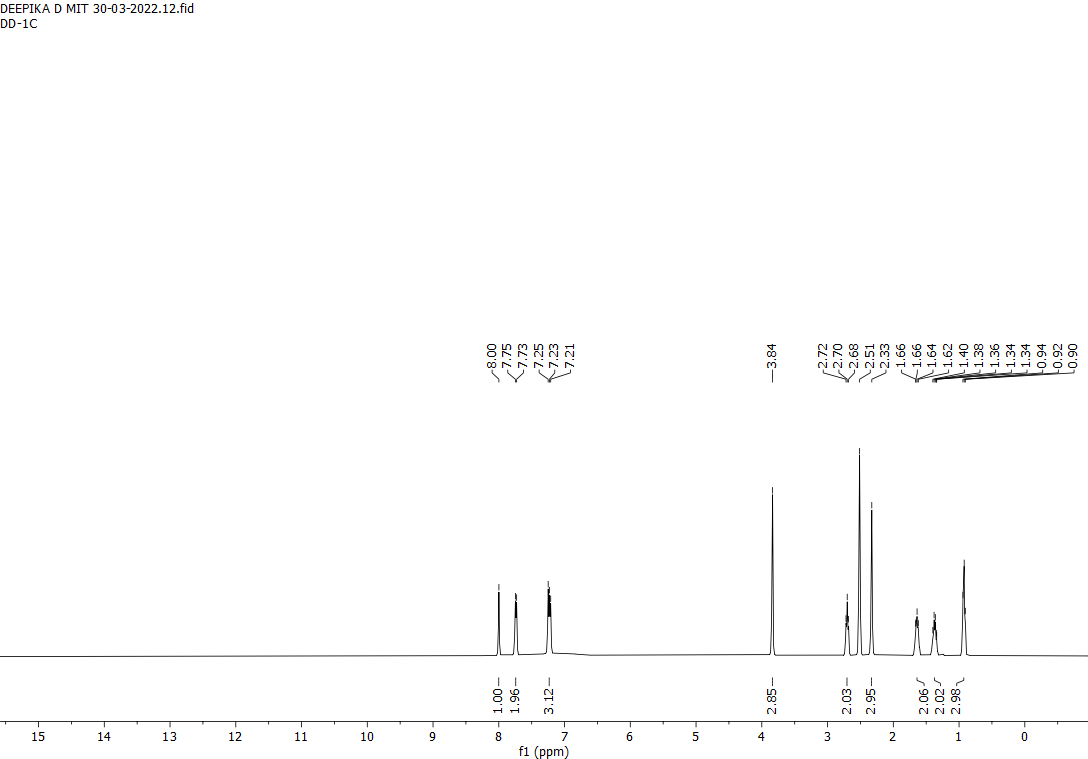


**Figure S9:** ^1^H NMR spectrum of **5c**


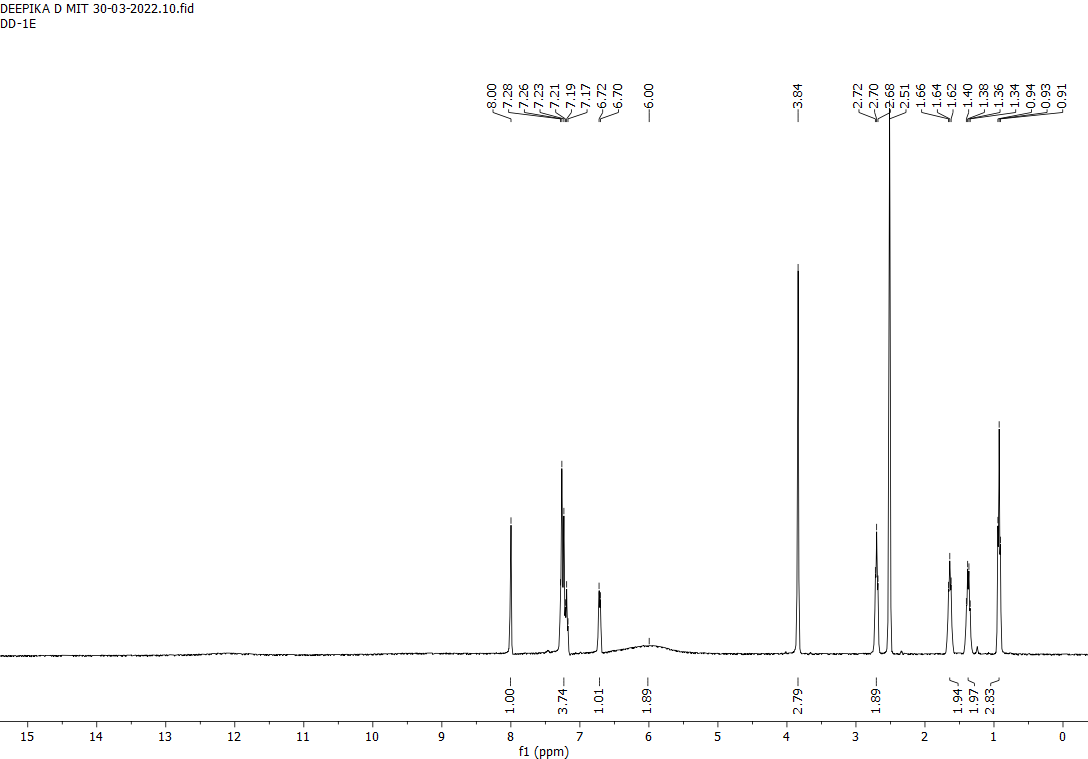


**Figure S10:** ^1^H NMR spectrum of **5d**


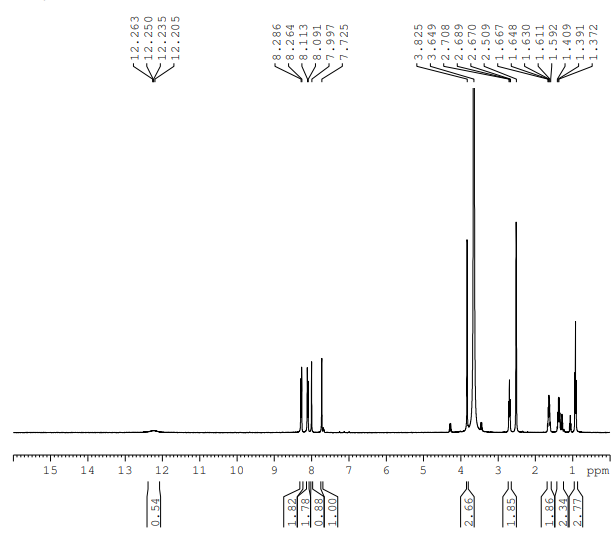


**Figure S11:** ^1^H NMR spectrum of **5e**

**
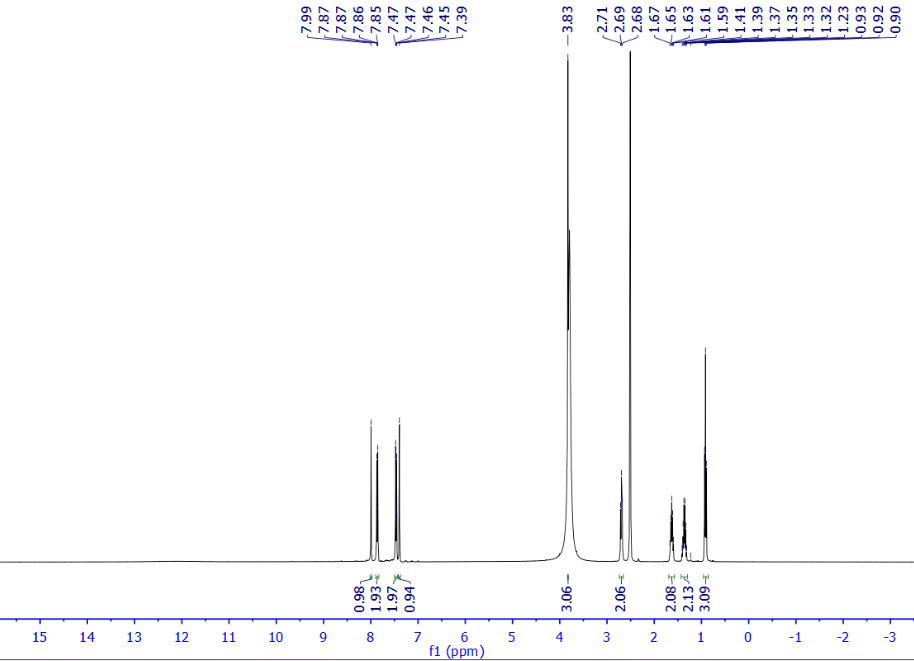
**

**Figure S12:** ^1^H NMR spectrum of **5f**

**
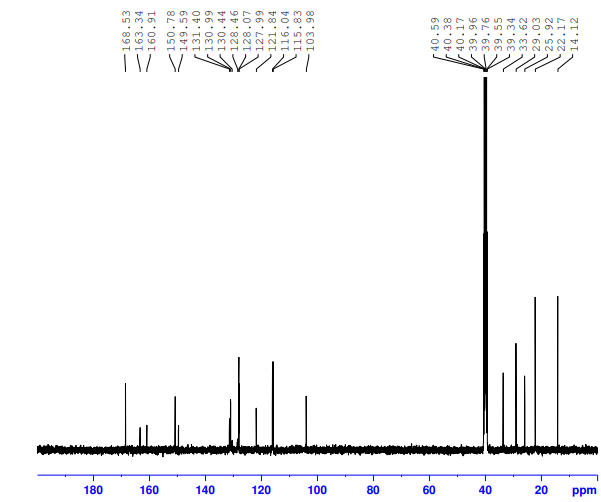
**

**Figure S13:** ^13^C NMR spectrum of **5a**

**
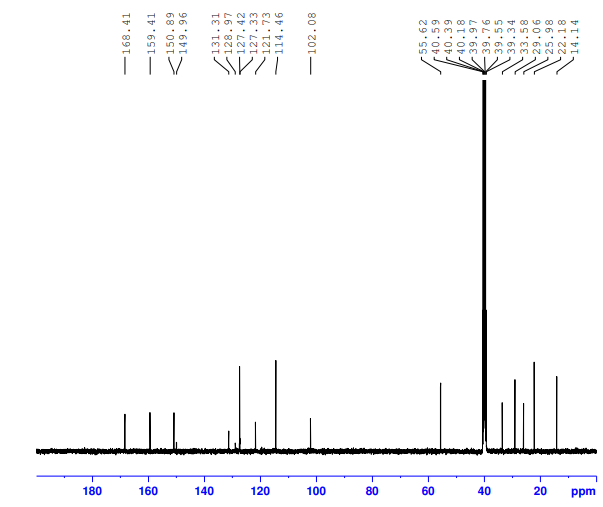
**

**Figure S14:** ^13^C NMR spectrum of **5b**

**
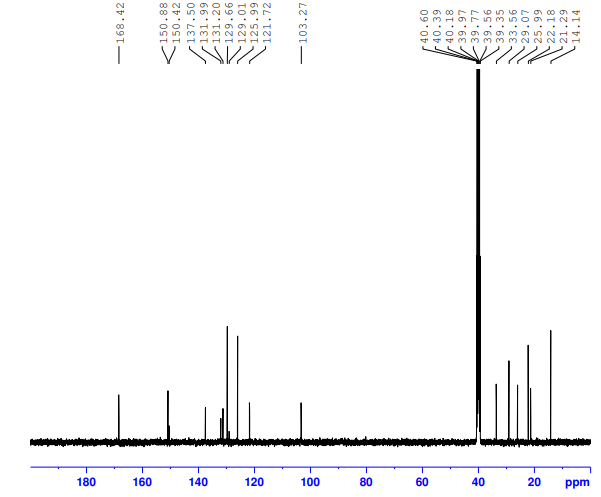
**

**Figure S15:** ^13^C NMR spectrum of **5c**

**
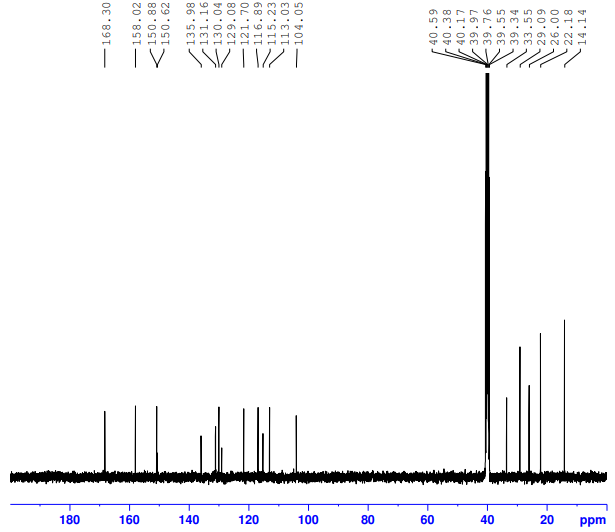
**

**Figure S16:** ^13^C NMR spectrum of **5d**

**
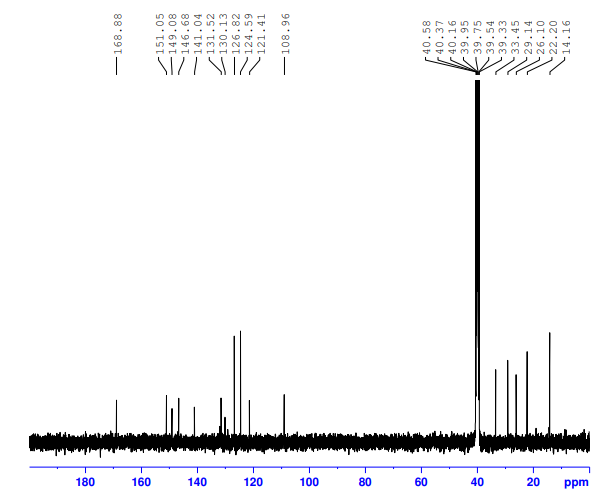
**

**Figure S17:** ^13^C NMR spectrum of **5e**

**
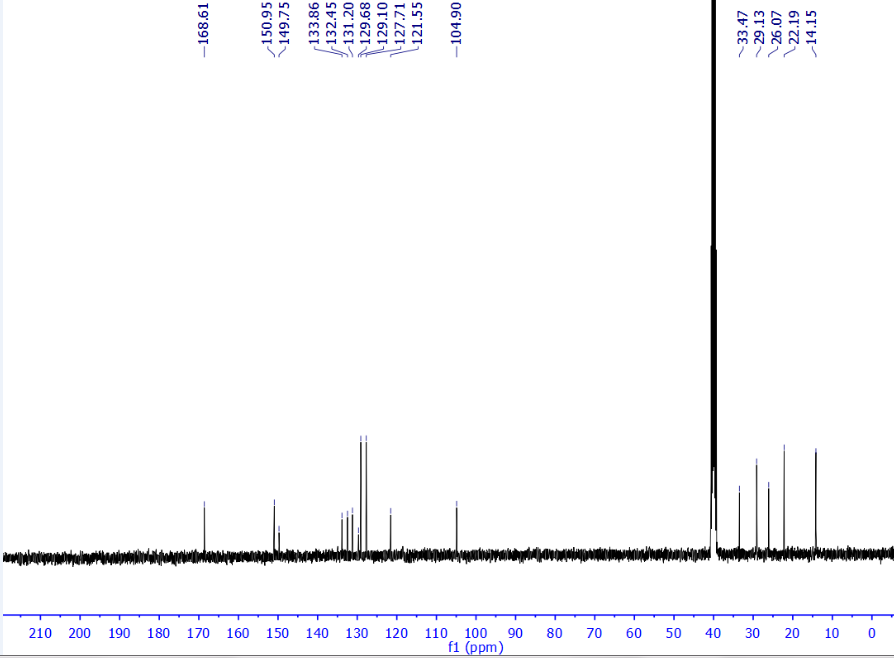
**

**Figure S18:** ^13^C NMR spectrum of **5f**

**Liquid Chromatography Mass Spectrometry (LC-MS)**

**
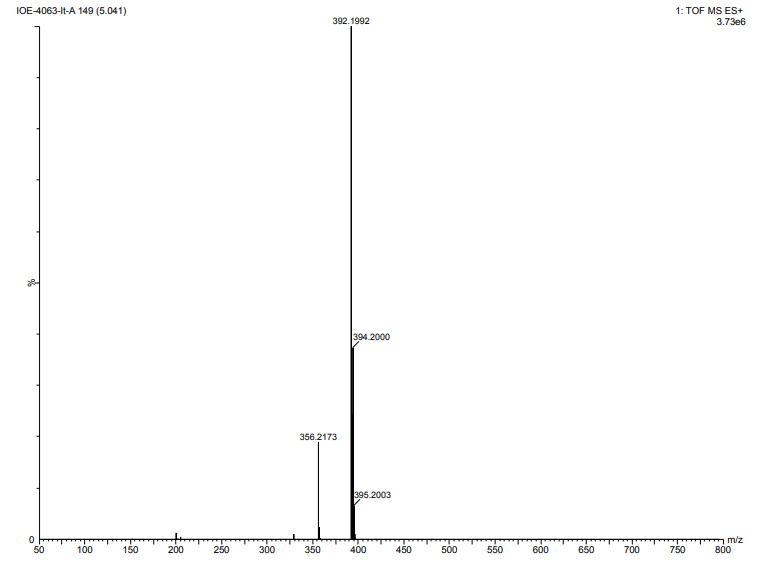
**

**Figure S19:** LCMS of **5a**

**
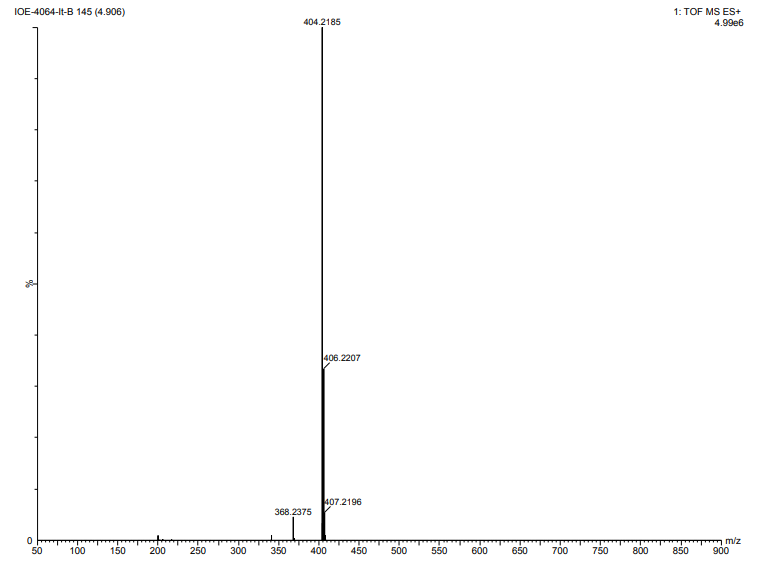
**

**Figure S20:** LCMS of **5b**

**
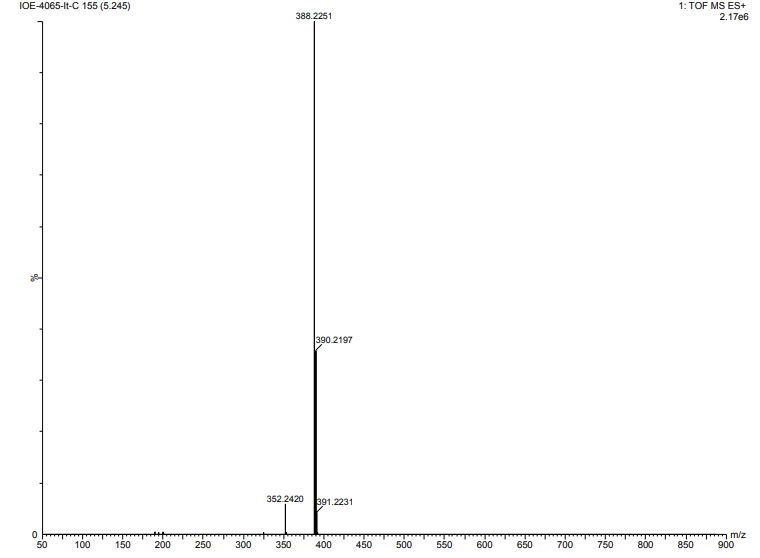
**

**Figure S21:** LCMS of **5c**

**
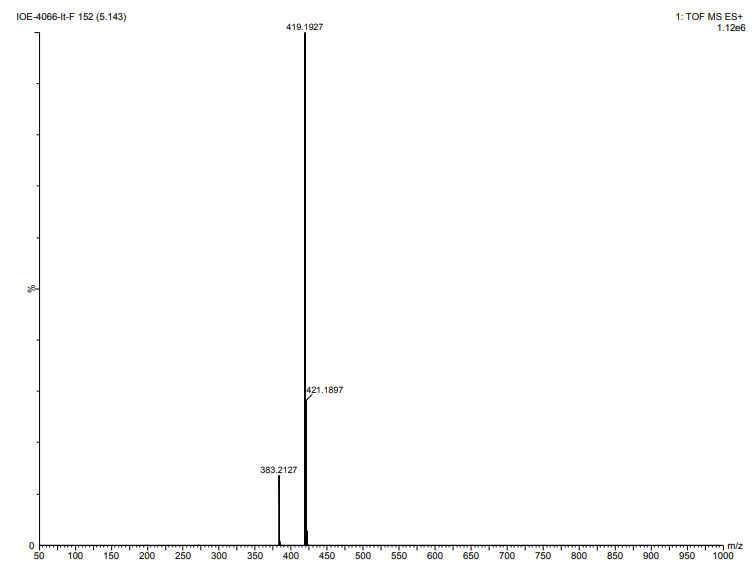
**

**Figure S22:** LCMS of **5e**

**
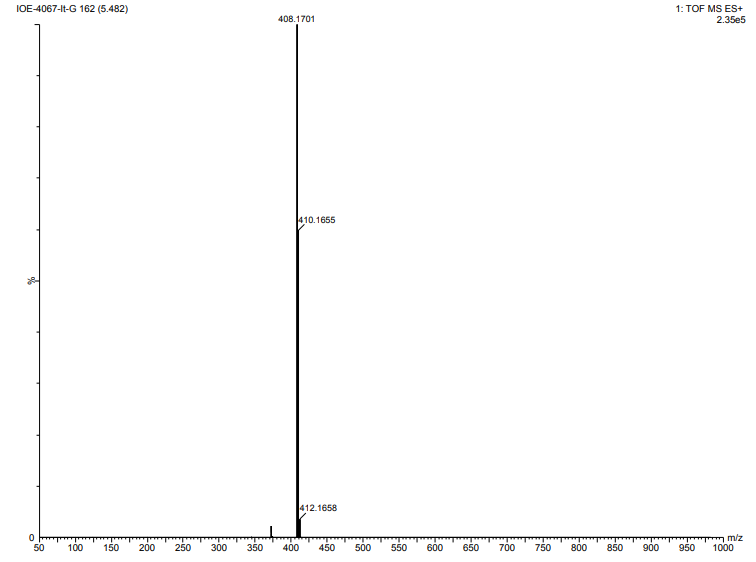
**

**Figure S23:** LCMS of **5f**
